# Best Practice Guidelines for the Management of Patients with Post-Stroke Spasticity: A Modified Scoping Review

**DOI:** 10.3390/toxins16020098

**Published:** 2024-02-10

**Authors:** Areerat Suputtitada, Supattana Chatromyen, Carl P. C. Chen, David M. Simpson

**Affiliations:** 1 Department of Rehabilitation Medicine, Faculty of Medicine, Chulalongkorn University, King Chulalongkorn Memorial Hospital, Rama 4 Road, Patumwan, Bangkok 10330, Thailand; 2 Principles and Practice of Clinical Research (PPCR) Program, Harvard T.H. Chan School of Public Health, Boston, MA 02115, USA; 3 Neurological Institute of Thailand, Department of Medical Services, Ministry of Public Health, Bangkok 10400, Thailand; dfondsupattana@gmail.com; 4 Department of Physical Medicine & Rehabilitation, Chang Gung Memorial Hospital at Linkou, College of Medicine, Chang Gung University, Guishan District, Taoyuan City 33343, Taiwan; carlchendr@gmail.com; 5 Department of Neurology, Icahn School of Medicine at Mount Sinai, New York City, NY 10029, USA; david.simpson@mssm.edu

**Keywords:** post-stroke spasticity, best evidence, GRADE A, a modified scoping review, multimodality treatments

## Abstract

This article aims to provide a concise overview of the best available evidence for managing post-stroke spasticity. A modified scoping review, conducted following the PRISMA guidelines and the PRISMA Extension for Scoping Reviews (PRISMA-ScR), involved an intensive search on Medline and PubMed from 1 January 2000 to 31 August 2023. The focus was placed on high-quality (GRADE A) medical, rehabilitation, and surgical interventions. In total, 32 treatments for post-stroke spasticity were identified. Two independent reviewers rigorously assessed studies, extracting data, and evaluating bias using GRADE criteria. Only interventions with GRADE A evidence were considered. The data included the study type, number of trials, participant characteristics, interventions, parameters, controls, outcomes, and limitations. The results revealed eleven treatments supported by GRADE A evidence, comprising 14 studies. Thirteen were systematic reviews and meta-analyses, and one was randomized control trial. The GRADE A treatments included stretching exercises, static stretching with positional orthosis, transcutaneous electrical nerve stimulation, extracorporeal shock wave therapy, peripheral magnetic stimulation, non-invasive brain stimulation, botulinum toxin A injection, dry needling, intrathecal baclofen, whole body vibration, and localized muscle vibration. In conclusion, this modified scoping review highlights the multimodal treatments supported by GRADE A evidence as being effective for improving functional recovery and quality of life in post-stroke spasticity. Further research and exploration of new therapeutic options are encouraged.

## 1. Introduction

Stroke remains the second-leading cause of death and the third-leading cause of death and disability combined in the world. From 1990 to 2019, its burden increased substantially, with the bulk of the global stroke burden residing in lower-income and lower-middle-income countries [1]. It is estimated that approximately one-third of post-stroke patients develop symptomatic spasticity [2]. Spasticity was originally defined by Lance in 1980 as a velocity-dependent increase in muscle tone resulting from the hyperexcitability of the tonic stretch reflex in patients with an upper motor neuron injury. If left untreated, spasticity can be painful, distressing, and a potential cause of disability [3].

In 2012, there was a topical review by Francisco et al. [4] on post-stroke spasticity management, which included a literature review on outcome measurements, goals for the management of post-stroke spasticity, and a proposed treatment algorithm that included botulinum toxin injection, neurolysis, oral drugs, intrathecal baclofen, and surgical intervention. Bavikatte et al. [5] mainly focused on the criteria for referral to specialists by using the “proposed traffic light system”. They also reviewed oral drugs, botulinum toxin alone or with shockwave therapy or with electrical stimulation, adhesive taping, and casting. 

However, most of the published guidelines mainly focus on post-stroke rehabilitation in general, while some of them only focus on botulinum toxin injection [3]. To the best of our knowledge, there is no comprehensive review of the best practice guidelines focusing on post-stroke spasticity. This scoping review addresses the latest updates on the best evidence available on oral medications, physical therapies, occupational therapies, botulinum toxin injection, neurolysis, newly innovated therapies, alternative medicines, and surgeries. This guideline is limited to spasticity alone in adults with post-stroke spasticity. Extraordinarily, we selected the treatments that proved to be high quality based on the GRADE (grading of recommendations, assessment, development, and evaluation) [6,7] approach. High-quality systematic reviews and clinical practice guidelines must now include GRADE, as it offers a systematic, clear, and explicit method for determining the certainty of the evidence and making recommendations for best practices. Recognizing that not all patients will respond to recommendations in the clinical practice guidelines in every situation is crucial. Individual patient conditions, preferences, and values must always be considered while using evidence-based medicine [7,8].

By implementing these top-quality treatments, patients can receive care that is based on the most current and reliable research. This strategy not only enhances confidence in medical interventions but also places patient outcomes as a priority, striving for the utmost standards of healthcare delivery.

Objective: The objective of this modified scoping review is to define the best evidence available for the management of spasticity to prevent complications arising in stroke patients.

## 2. Results

### 2.1. Description of Included Studies

**Figure 1:**
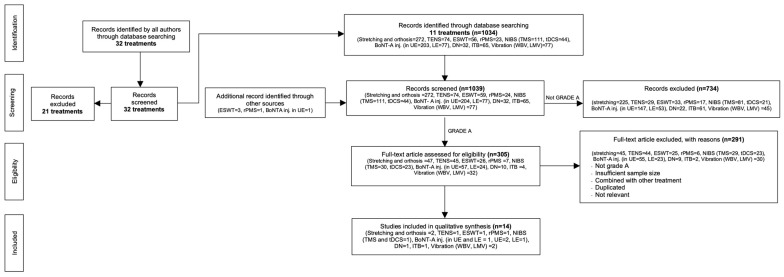
PRISMA flow chart modified by Areerat Suputtitada (AS). TENS: transcutaneous electrical nerve stimulation; ESWT: extracorporeal shock wave therapy; rPMS: repetitive peripheral magnetic stimulation; NIBS: non-invasive brain stimulation; TMS: transcranial magnetic stimulation; tDCS: transcranial direct current stimulation; BoNT-A inj.: botulinum toxin A injection; DN: dry needling; ITB: intrathecal baclofen; UE: upper extremity; LE: lower extremity; WBV: whole-body vibration; and LMV: localized muscle vibration.

The database searches retrieved 11 interventions of GRADE A evidence (out of the 32 listed interventions). A scoping review of the latest articles that are high quality or “A” by GRADE [6,7] (i.e., systematic reviews, meta-analyses, and randomized control trials) regarding the treatments for post-stroke spasticity included in Figure 1 was performed according to the Preferred Reporting Items for Systematic Review and Meta-Analysis (PRISMA) guidelines [9] and the PRISMA Extension for Scoping Reviews (PRISMA-ScR) [10]. The search flow for each topic is shown with reasons for exclusion in Figure 1: PRISMA flow chart modified by AS. The details of PRISMA-ScR are in the Supplementary file titled “Supplementary File S1-PRISMA”.

### 2.2. Study Characteristics

**Figure 2:**
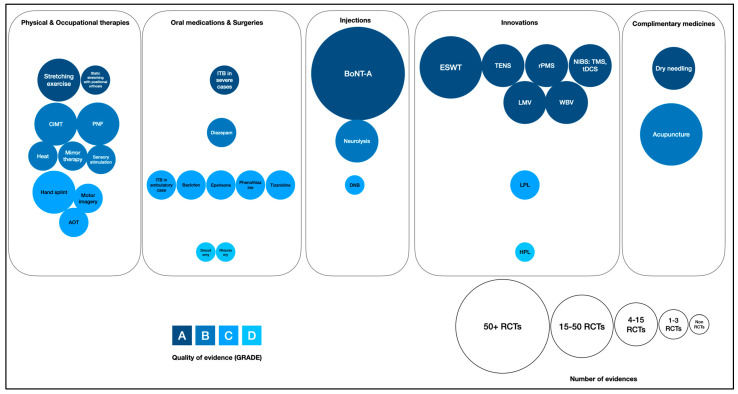
**State of the evidence (packed bubble chart)**. CIMT: constraint-induced movement therapy; PNF: proprioceptive neuromuscular facilitation; AOT: action observation therapy; ITB: intrathecal baclofen; BoNT-A: botulinum toxin A; DNB: diagnostic nerve block using anesthetic products; ESWT: extracorporeal shock wave therapy; TENS: transcutaneous electrical nerve stimulation; rPMS: repetitive peripheral magnetic stimulation; NIBS: non-invasive brain stimulation; TMS: transcranial magnetic stimulation; tDCS: transcranial direct current stimulation; WBV: whole-body vibration; LMV: localized muscle vibration LPL: low-power laser; and HPL: high-power laser.

Of the eleven treatments, fifteen studies with GRADE A evidence were collected. Thirteen studies were systematic reviews and meta-analyses, and one was randomized control trial. The included characteristics of each intervention and study are presented in Table 1. All 32 interested treatments were presented in packed bubble chart format, modified from the Evidence Alert System adapted from the “State of the Evidence Traffic Lights 2019: Systematic Review of Interventions for Preventing and Treating Children with Cerebral Palsy” [11], as shown in Figure 2. We used the PICO [12] to establish the inclusion criteria for this review as detailed in the Supplementary file titled “Supplementary File S2-Risk of bias”.

### 2.3. Included Interventions

#### 2.3.1. Stretching Exercise

Summary: It is suggested that passive static/dynamic stretching should be considered an adjunctive therapeutic approach in the management of post-stroke spasticity. However, it is important to note that the cumulative daily duration of such a form of stretching should not exceed 2.5 h to avoid the risk of pain.

Highlighted details: Passive stretching is the main physical therapy method for reducing the level of spasticity after a stroke, but it is often used with other therapies as well. In 2021, Gomez et al. [13] conducted a meta-analysis on how well different kinds of stretching work when carried out on their own. Long-term studies incorporating stretching with orthotic devices or other forms of rehabilitation were not considered. Only six randomized controlled trials with fair-to-good methodological quality were considered. Interventions lasted anywhere from a few minutes to an hour or more per session across the studies. They concluded that a stretching intervention alone has not been proven to be effective; non-statistically significant results in favor of the intervention group were observed for the Modified Ashworth Scale (MAS). In conclusion, passive static/dynamic stretching could be carried out periodically but must be performed in conjunction with other rehabilitation treatments. Constraints and other forms of passive static stretching should be avoided in favor of more frequent, shorter, and more intense repetitions. The time for rest between sets should be at least 60 min, and the total time for stretching each day must be no more than 2.5 h to avoid pain [28].

#### 2.3.2. Static Stretching with Positioning Orthoses

Summary: The effectiveness of static stretching with wrist devices to reduce wrist flexor spasticity in chronic stroke patients is still uncertain. Further research with larger sample sizes is needed.

Highlighted details: Orthotic positioning and stretching of the affected muscles are common treatments for spasticity. However, the therapy’s efficacy, optimal frequency, intensity, and duration are uncertain. In 2018, Salazar et al. [14] published a systematic review that included three low-quality (by the GRADE system) RCTs (57 patients in total) and suggested that static stretching with wrist devices in a neutral or extended wrist position might be superior to no therapy to reduce wrist flexor spasticity in chronic stroke patients. Due to factors such as the low sample size and high levels of heterogeneity, caution is warranted in drawing conclusions from these findings (despite the similar splinting protocol). Over the course of three to four weeks, patients wore positioning orthoses for six to seven days a week, stretched for twenty to forty-five minutes a day (spread out over the course of two or three sessions), and participated in other intervention techniques. Although positioning orthoses are effective in reducing wrist flexor spasticity in clinical practice, prolonged orthosis use is not encouraged. A randomized controlled trial [29] found that wrist splinting in the neutral or extended position for more than 9 to 12 h per day for 4 weeks did not reduce wrist contracture.

#### 2.3.3. Transcutaneous Electrical Nerve Simulation (TENS) as an Adjuvant Therapy

Summary: High-frequency TENS, as an adjuvant therapy, can be beneficial in reducing post-stroke spasticity in the lower extremities.

Highlighted details: Studies on the effects of TENS on spasticity in adults following a stroke have been published in recent years. In 2018, Mahmood et al. [15] published a comprehensive review and meta-analysis of the relevant literature. TENS (as an adjunct therapy) was effective in reducing spasticity in the lower limbs of long-term stroke survivors. Because TENS is a low-cost, self-administered treatment with fewer side effects than other therapies, this study is very applicable in the clinical setting. Stretch reflex excitability reduction, increased presynaptic inhibition, and modulation of reciprocal inhibition are all possibilities. High-frequency TENS (100 Hz), lasting more than 30 min per session, electrode placement along the nerve or the muscle belly, intensity twice that of the sensory threshold, and treatment lasting more than 2 weeks are all recommended. However, the effect on upper limb spasticity should be further investigated because only two RCTs studied the effect of TENS on reducing spasticity in the upper limbs. Three studies reported that the effects could last for 2–5 weeks, whereas two studies reported that the effects were maintained for less than a day and the spasticity returned to baseline after the intervention ended. The patient should be made aware of this discrepancy in these findings before any treatment is administered. Due to a lack of research examining the impact of low-frequency TENs on spasticity, it is still unclear whether high- or low-frequency TENS is preferable. Although Marcolino et al. [30] found no statistically significant differences between the high- and low-frequency treatment protocols in a subgroup analysis, more research comparing high- and low-frequency TENS is warranted.

#### 2.3.4. Extracorporeal Shock Wave Therapy (ESWT)

Summary: Radial ESWT can be effective in reducing post-stroke spasticity, and the effects of this treatment can last for 12 weeks.

Highlighted details: In recent years, clinical studies have demonstrated that ESWT can be used to treat spasticity after a stroke. Duan et al. [31] compiled potential ESWT mechanisms of interest. First, ESWT stimulates the production of nitric oxide, following which it reduces the level of acetylcholine at the neuromuscular junction, thereby alleviating muscle spasms. Second, by vibratory stimulations of the tendons, ESWT may reduce the excitability of motor neurons, but this effect is not long-lasting. Third, ESWT involves transient nerve conduction dysfunction at the neuromuscular junction, but the number of receptors at the NM junction will recover at an extremely rapid rate, which can explain the short duration of the shock wave effect in reducing spasticity in some studies. Finally, tensile stress and shear force from ESWT can induce tissue release, improve the microcirculation of muscles, and thereby alleviate the condition of muscle spasm. Zhang et al. [16], in 2022, published a systematic review and meta-analysis evaluating the efficacy of ESWT on spasticity following an upper motor neuron injury. This systematic review included 42 studies with 1973 patients, and 34 studies were included in the meta-analysis. Twenty-nine studies involved stroke (multiple sclerosis: one study, spinal cord injury: one study, and cerebral palsy: eleven studies). Based on the subgroup analysis, radial ESWT was superior against focused ESWT in relieving spasticity, possibly due to a larger therapeutic area and higher energy in superficial tissue. Higher pressure and frequency produced better results (6 Hz). The effect lasted for a month following the treatment, but a single session of ESWT had no significant effect on the MAS score. This indicated that repeating ESWT within four weeks of the previous treatment may be beneficial for effect maintenance. Because patients and interventionists could not be blinded with this type of treatment, there is a risk of bias in the included studies. The conclusions of this systematic review and meta-analysis are consistent with those of prior research [32,33,34,35,36,37,38].

#### 2.3.5. Repetitive Peripheral Magnetic Stimulation (rPMS)

Summary: rPMS may be beneficial in post-stroke spasticity, but there are no conclusions regarding the optimal protocol and duration of the treatment.

Highlighted details: rPMS is a non-invasive and painless method with negligible side effects. It can generate a magnetic field that stimulates the peripheral nerves and muscles. The potential mechanism may involve the neuromodulation effect of rPMS when applied to muscles or nerves. Pan et al., 2022 [17], conducted a systematic review of eight randomized controlled trials (RCTs) and a meta-analysis of six trials involving 170 patients with chronic stroke (>6 months). When evaluated with the AS or MAS, the results indicated that rPMS has the potential to reduce spasticity in the upper and lower extremities. The parameters used in the trials were variable and ranged from 5 to 25 Hz in frequency, 3 to 30 min per session, and a round or figure-eight coil type. There was no investigation into the duration of the treatment effect. However, additional research with a larger sample size is required to determine the optimal protocol and duration of the outcome.

#### 2.3.6. Non-Invasive Brain Stimulation (NIBS)

Summary: Low-frequency repetitive transcranial magnetic stimulation (rTMS) of an unaffected hemisphere and/or anodal transcranial direct current stimulation (tDCS) of an affected hemisphere can be effective in reducing post-stroke spasticity in the upper extremities.

Highlighted details: NIBS, such as rTMS and tDCS, has received considerable attention in recent years. High-frequency stimulation (5 Hz or higher) increases cortical excitability, whereas low-frequency stimulation (1 Hz or below) decreases cortical excitability during rTMS. For tDCS, a weak direct current modulates the activity of cerebral cortex neurons. While anodal tDCS increases cortical excitability, cathodal tDCS decreases it. The most recent systematic review and meta-analysis conducted by Wang et al. in 2022 [18] revealed moderate evidence that NIBS can reduce spasticity after a stroke. The ability of NIBS to alter the excitability of the cerebral motor cortex and indirectly decrease the excitability of motor neurons in the spinal cord via the H-reflex is one possible mechanism. Eleven RCTs demonstrated that rTMS has significant benefits for patients with post-stroke spasticity in the upper limbs, but only low frequency rTMS applied to stimulate the unaffected hemispheres has significant benefits. Seven RCTs on tDCS were included in the meta-analysis; however, only anodal stimulation with 0.7 mA or 1.2 mA significantly reduced upper limb spasticity (not 2.0 mA). It is thought that anodal tDCS stimulation on the affected side can increase the excitability of the affected cortex. Further studies on lower limb spasticity, investigation of mechanisms, and demonstration of the duration of efficacy are still needed. In addition, other forms of NIBS, such as transcranial alternating current stimulation (TACS) and transcranial ultrasound stimulation (TUS), are not supported by sufficient evidence.

#### 2.3.7. Botulinum Toxin A (BoNT-A) Injection 

Summary: BoNT-A is essential for the management of post-stroke spasticity, as there is compelling evidence that it effectively reduces spasticity. Nevertheless, the extent to which it contributes to functional recovery is still an issue of debate. Optimal timing is essential, as injections that specifically target the flexor muscles within a period of 4–6 weeks after a stroke have been shown to be the most effective. The benefits of BoNT-A include its capacity to regulate cortical excitability, thereby mitigating maladaptive plasticity and preventing contractures. Ultrasound (US)-guided injections improve safety and accuracy, with ergonomics playing a crucial role in maximizing results. Administering a large amount of OnaBoNT-A (600 units or more) is considered safe for treating severe spasticity. However, its effects on functionality and spasticity-related pain are still uncertain, indicating the need for additional research.

Remarks:

1. The injection dose may be modified based on the patient’s age, body weight, muscle mass, and effectiveness.
2. We agreed to lower the dose for patients in hot-climate countries or with small muscle mass [39].
3. The injection intervals should be 12 weeks or more.
4. The maximum injection doses accepted are as follows:
  - Botox^®^ (OnaBoNT-A) injections range from 5 to 100 units per muscle, with a maximum dose of 400 units per visit [40].
  - Dysport^®^ (AboBoNT-A) injections range from 100 to 250 units per muscle, with a maximum dose of 1500–2000 units per visit. In small- and medium-volume muscles, the mean/median dose often varied between 100 U and 200 U when only values for 50 or more treated patients were taken into account. The same pattern was noted for the muscle group with a significant volume; however, the average/middle AboBoNT-A dosage was more inclined to range from 200 U to 500 U, especially in larger muscles [19].
  - Xeomin^®^ (IncoBoNT-A) injections range from 5 to 100 units per muscle, with a maximum dose of 400 units per visit.
5. In available settings, US -guided BoNT-A injections can be useful, especially in distal upper extremities’ muscles, such as wrist and finger flexors.

Highlighted details: The development of BoNT-A is driven by the consequences of post-stroke spasticity. The efficacy of it in lowering spasticity is supported by high-quality GRADE A evidence; however, there are ongoing arguments about its role in functional recovery [22,41,42]. The significance of timing is emphasized by our experience since injections that target the flexor muscles in the fingers and wrists within 4 to 6 weeks after a stroke are found to be most effective [43]. The possible effects of BoNT-A include mitiga- ting maladaptive plasticity and avoiding contractures via modulating cortical excitability [44]. The utilization of US guidance for providing BoNT-A injections offers several advantages. These include the ability to visualize the needle’s trajectory in real time, thereby preventing harm to vital tissues, particularly with in-plane guidance. Additionally, US guiding allows for the precise localization of the target muscle groups [45,46]. Selection of key muscles to be injected. Decrease dosages for patients who reside in hot climates where heat can affect muscular flexibility [39,47]. Personalized rehabilitation programs designed to meet the specific needs of each patient after receiving injections are crucial for optimizing their functional outcomes [47,48]. US -guided BoNT-A injections for muscle spasticity are increasingly popular and essential. Lagnau P et al. [46] underscored the significance of US ergonomics in BoNT-A injections and offered expert recommendations. The lack of awareness of ergonomics for US-guided BoNT-A injections may lead to suboptimal patient outcomes, increased work-related injuries, and patient discomfort. 

Spasticity-related pain (SRP) is common and can lead to restrictions in functionality and debilitating effects. Several publications have indicated that greater doses of BoNT-A, above the initial recommendations, can be utilized with efficacy and safety, particularly in cases of severe spasticity. However, it remains uncertain whether this treatment has any advantages in terms of its functional results and SRP. The term “high dosage” refers to a quantity of 600 units or more of OnaBoNT-A and IncoBoNT-A. There is a lack of empirical evidence to definitively demonstrate that this therapy approach enhances the functionality of the limbs. While there is no definitive proof, some patients with spasticity may have targeted relief from high-dose BoNT-A. Similarly, there is not enough data to suggest a high BoNT-A dosage in SRP [49]. 

The comprehensive meta-analysis with a broad range of characteristics by Sun in 2019 [20] showed that BoNT-A injections could significantly improve muscle tone in upper limb spasticity compared with the placebo group. In conclusion, BoNT-A injections are effective and safe in reducing spastic symptoms and improving hygiene care for at least 12 weeks. This finding is consistent with previous studies by Dong et al. [50] in 2017. A lower dose is recommended in preference to a higher dose for increasing the functionality of the upper extremities, especially in hot-climate countries, according to Suputtitada et al. [39] One systematic review and meta-analysis by Andringa A et al. [21] reported that robust evidence is shown for the effectiveness of BoNT-A in reducing the Modified Ashworth Score and improving the self-care ability of the affected hand and arm after the intervention and at follow-up. They also concluded that from 40 trials, including 2718 stroke patients, no further trials are needed to investigate BoNT-A injections for their favorable effects. Furthermore, in available settings, US -guided BoNT-A injections can be useful, especially in distal upper extremity muscles such as the wrist and finger flexors [51].

According to Doan et al., 2021 [22], in patients with lower extremity spasticity following a stroke, BoNT-A injections are recommended, measured with the Modified Ashworth Scale (MAS). The doses of approximately 300 units of Botox^®^ and 1000 units of Dysport^®^ appear to be the most favorable for spastic plantar flexors. Furthermore, in adults with spastic equinus, US -guided injection allows for completely accurate injection by precisely evaluating the needle position and muscle thickness, while needle placement into the gastrocnemius of adults with spastic equinus is not completely accurate when guided by palpation or electrical stimulation (ES) [52]. However, more studies are needed, especially to prove active function gain. 

The results of the meta-analysis emphasize better outcomes and encourage doctors to use imaging or electrophysiological techniques to direct BoNT-A injections in people with limb spasticity. Within electrophysiological options, ES outperforms electromyography (EMG), especially when considering patient comfort. The decision between US and ES depends on the equipment that is available, the discomfort of the patient, and the practitioner’s background. US is useful because it provides real-time imaging and little discomfort, especially in academic hospitals that treat patients with unusual spasticity symptoms. It is advised for physicians with less training to begin with ES and move on to US. The selection of the best technique is heavily influenced by the patient’s comfort and compliance, underscoring the significance of individualized, patient-centered strategies in clinical practice [53].

#### 2.3.8. Dry Needling (DN)

Summary: DN can be useful as an adjuvant therapy and should be combined with rehabilitation therapy to reduce post-stroke spasticity only in the lower extremities at short-term follow-up (1 week), but is not effective when evaluated at 4 weeks [23].

Highlighted details: This meta-analysis [23] analyzed the impact of muscle DN on post-stroke patients. Very low to moderate evidence indicates that DN has a positive effect on reducing spasticity (muscle tone) in stroke survivors. No effects on motor function were observed. However, pooled data from the present meta-analysis did not show significant effects on spasticity in the upper extremities. Most trials investigated short-term effects (1 week), with only two studies investigating longer follow-up periods (4–6 weeks). Therefore, randomized clinical trials examining the long-term effects of DN in post-stroke patients are needed. Previous studies used techniques such as DN over the most painful spot within a spastic taut band, targeting active trigger points (reproducing the refer pain), and standardizing belly muscle points.

#### 2.3.9. Intrathecal Baclofen (ITB)

Summary: ITB therapy can be used as an alternative to conventional medical management for the treatment of generalized, severe post-stroke spasticity in adults.

Highlighted details: ITB therapy is known as an option for use in severe, chronic spasticity of cerebral or spinal origin, including generalized spastic hypertonia following stroke. However, evidence for its effectiveness in the treatment of spasticity mainly comes from controlled studies in cerebral palsy and multiple sclerosis. SISTERS (Spasticity In Stroke–Randomized Study) [24] was a multicenter, open-label randomized controlled trial to evaluate the efficacy and safety of ITB therapy versus conventional medical management (CMM) with oral antispastic medications in sixty participants with generalized, severe post-stroke spasticity (chronic stroke with spasticity in ≥2 extremities and an Ashworth Scale (AS) score ≥ 3 in at least two affected muscle groups in the lower extremities). There was a significant effect of treatment with ITB therapy over CMM for reduction of spastic hypertonia and muscle tone in the lower limb of the affected side over a 6-month follow-up [25]. Reduction in pain scores, improvement in quality-of-life measures, and high patient satisfaction with therapy were also demonstrated as secondary outcomes. More patients reported adverse events while receiving ITB (24/25 patients, 96%; 149 events) compared with CMM (22/35, 63%; 77 events), although these were generally consistent with the known safety profile of ITB therapy. Future studies with larger sample sizes, longer follow-up, and cost-effectiveness analysis are required.

#### 2.3.10. Whole-Body Vibration Therapy (WBV)

Summary: WBV is supported by moderate-quality evidence as an adjunct treatment for spasticity in patients, particularly beneficial for individuals under the age of 60 years when applied at frequencies below 20 Hz for a duration of 10 min.

Highlighted details: The systematic review and meta-analysis by Zhang et al. [26] critically assessed the impact of WBV on post-stroke spasticity. It analyzed 11 RCTs with a total of 475 patients, all of which were published in either English or Chinese. This meta-analysis concluded that WBV, specifically at frequencies lower than 20 Hz and durations of 10 min, effectively decreased spasticity in individuals under the age of 60 with acute and subacute stroke. Patients with chronic stroke had insufficient evidence to support a substantial decrease in spasticity compared to the control groups. These studies demonstrated that WBV, when combined with other treatments, was more successful than control treatments alone. When used in conjunction with other therapies, WBV has been shown to produce better results for upper limb spasticity. A significant discovery revealed that vibration frequencies below 20 Hz were more effective in decreasing spasticity, and the optimal period of vibration was determined to be 10 min. The present meta-analysis does possess limitations, such as the inclusion of individuals with only mild to moderate severity of spasticity and the absence of a comprehensive study on instances of severe spasticity. Moreover, the complete evaluation of the potential long-term advantages of WBV is hindered by the scarcity of studies that have conducted follow-ups lasting 3–6 months. Subsequent investigations should endeavor to examine the impacts of intense spasticity and validate the enduring effectiveness of this intervention.

#### 2.3.11. Localized Muscle Vibration (LMV)

Summary: LMV is proposed to be a viable and safe adjunct to conventional neurorehabilitation programs to reduce post-stroke spasticity. Further studies, with a larger number of homogeneous patients and a shared methodology, are needed to produce more reliable data, especially on the lower limbs.

Highlighted details: A recently published systematic review [27] by Avvantaggiato et al. consolidates the evidence on the efficacy of LMV in treating spasticity in post-stroke survivors. This review contained a total of 14 RCTs with 425 participants. All of the studies except one (uncertain risk of selection, performance, and detection bias) were judged to be of high or moderate quality. This review revealed a statistically significant reduction in the MAS in the elbow (*p* = 0.001; I^2^ = 0%) and wrist (*p* = 0.04; I^2^ = 36%) but not in the shoulder (*p* = 0.26; I^2^ = 0%). The analysis indicates that integrating LMV into traditional rehabilitation approaches may enhance the outcomes related to spasticity management. It stands out as a non-invasive modality that can be safely administered alongside other treatments. However, this review also acknowledges the limitations within the field of study. Since post-stroke motor impairments frequently have a significant impact on the lower extremities, there is a lack of research on this topic. It is also hard to figure out the best LMV protocols because different studies have different types of vibrations, different lengths of sessions, different frequencies, different locations of spasticity, and different control treatments. This variability impedes the ability to draw definitive conclusions regarding the most efficacious application of LMV. Additionally, the current body of evidence does not provide conclusive insights into the longevity of its effects. This highlights the significance of conducting further research to ascertain the duration of the advantages of LMV and determine the optimal treatment settings. Such investigations will enhance the utility and efficacy of this therapy.

## 3. Discussion

Post-stroke spasticity is still a problem that can hinder a person’s ability to recover and function. New treatment modalities have significantly expanded the treatment team’s options. It is essential for the physician to collaborate with other clinicians, patients, and their caregivers to develop a comprehensive treatment plan with attainable and significant objectives. With the rapid development of new treatments, even longer periods of recovery are possible with greater improvements. When selecting treatment interventions, clinicians should be creative and, in collaboration with their patients, strive for a greater functional recovery [51].

In the treatment of post-stroke spasticity, a step-by-step strategy has been the standard for decades [54]. Reducing noxious stimulation is the first step in any program. Then, proper positioning and splinting would be applied to every patient as a required treatment. The subsequent step involved a pharmaceutical treatment. The choice of medication depended on disease severity, anatomical distribution, the presence of comorbidities, and the cost of the medication [54]. In focal spasticity, for instance, phenol/alcohol neurolysis or botulinum toxins would be chosen; in generalized spasticity, mild-to-moderate cases are typically treated with oral medications; if these fail, or in cases of severe generalized spasticity, intrathecal baclofen or surgical intervention may be considered in the available setting [54]. In practice, however, the clinician typically chooses the treatment based on his or her own competencies. For instance, a chemo-neurolysis-trained physician is likely to perform a nerve block. Less-confident physicians may be more likely to recommend splinting and/or oral medications. Due to a lack of high-quality research, physical modalities were rarely used.

Our modified scoping review included 11 interventions for post-stroke spasticity treatment, with 14 studies qualifying as the best evidence (high quality/GRADE A). The results demonstrated that multimodality treatments can also be used to improve functional recovery and quality of life; it is not mandatory to use a stepped approach, as was used for many years [54]. We have reviewed each intervention’s mechanism for post-stroke spasticity, including its efficacy, adverse effects, and duration of its benefits. These data assist in guiding the use of one or several of these treatments based on a holistic approach, such as the patient’s conditions, underlying diseases, and spasticity severity. For instance, patients with a needle phobia can benefit from ESWT instead of a botulinum toxin injection. Chronic post-stroke survivors with spasticity who suffer from depression may be candidates for NIBS. 

TENS offers the benefit of low cost and can be self-administered at home by either caregivers or patients. Home-use anodal tDCS can be applied when the patient is discharged to prolong TMS during admission. However, stretching exercises are still highly effective for maintaining range of motion. Combining botulinum toxin injections with stretching exercises can reduce the non-neuronal factors in spasticity. Moreover, in severe cases with an inadequate response to botulinum toxin injection, intrathecal baclofen (ITB) [24,25] is a promising intervention. According to a study by Francisco et al. [55], ITB can also benefit ambulatory stroke spasticity patients.

Despite these recommendations, the interventions that have been reviewed in this study should be used with some precautions. The study of stretching exercise, static stretching with positional orthosis, and dry needling still has small sample sizes. There is still limited research on the optimal parameter in TENS and rPMS. More data on the duration of efficacy in TENS, rPMS, NIBS, and DN are needed. ESWT has a well-designed study with many participants, but in a clinical setting, the outcomes may differ from the research results. There may be a variation in energy intensities from different shock wave devices and the patient’s condition, such as tolerance to the pain during ESWT treatment, skin lesions, or skin pressure tolerance. 

A redefined description of post-stroke spasticity and its influence on motor recovery throughout the transition from acute to chronic phases highlights that the stroke-induced aberrant neuroplasticity leads to the development of spasticity and motor-related deficits [56]. Spasticity does not have an immediate effect on functional recovery, but it obstructs genuine motor recovery by causing abnormal movement patterns and weakening of the muscles. The process of recovery is made more complex by factors such as abnormal force regulation, the simultaneous activation of many muscles, and the connection between different limbs. To manage spasticity, one must utilize approaches such as modifying mechanics and participating in neuromuscular reeducation. Although there may not be a direct causal relationship between reducing spasticity and achieving functional recovery, effectively controlling spasticity can enhance motor function throughout the long-term phase following a stroke. Acquiring a thorough comprehension of this interaction is crucial for optimizing the efficacy of rehabilitation efforts.

It is thought that sensitization may be a contributing factor to the development of muscular spasticity [57]. Promising advancements in the field of medical treatment, such as ESWT, rPMS, and DN, have their demonstrated potential for reducing spasticity and enhancing motor function. These treatments target the underlying causes of sensitization, including alterations in neurotransmitters, increased sensory inputs, tissue softness, and abnormalities with neuromuscular transmission. Nevertheless, the significant variability seen underscores the necessity for more careful investigations to validate these findings. Moreover, additional investigations are required to comprehensively comprehend the mechanics, long-term advantages, and potential hazards of these treatments.

Oral medication studies [58] do not reach the GRADE A level of evidence. Acute-to-subacute post-stroke patients usually suffer from multiple diseases and multiple drug exposures. There is some evidence suggesting that oral medications prescribed during the acute phase may limit the level of functional recovery during the acute phase. For example, benzodiazepines have been shown to reduce muscle tone, but they can also cause sedation and cognitive impairment, which can negatively impact a patient’s functional recovery [59]. Therefore, prescribing too many oral medications may not be the only option.

We recommend using any one of the best evidence-based treatments in this review alone or in combination as a multimodal therapy, depending on the clinical manifestations of the patient, such as the severity of spasticity or other diseases, the patient’s perspective and preferences, and so on.

### Strengths and Limitations

This scoping review systematically maps the literature on post-stroke spasticity. It was performed following the current guidance for scoping reviews [9].The methodological quality of the included studies was appraised using design-specific tools and provided insights into the quality of the literature within the field. Because many interventions were listed by multidisciplinary authors, the original versions of scoping reviews or systematic reviews may not be appropriate in this situation. Therefore, the modified version of the scoping review was developed to systematically incorporate multiple interventions in post-stroke spasticity. However, our search strategy included only one database, and as a result, we may have missed relevant GRADE A studies. In addition, we have analyzed and detailed all the risks of bias stated in the systematic review and meta-analysis studies and the randomized control trial we selected in our scoping review and put the authors’ perspectives in the Supplementary file titled “Supplementary File S2-Risk of bias”.

Randomized controlled trials (RCTs) are considered the gold standard of design but may not always be feasible in PRM research, especially for non-pharmacological interventions. Difficulties in implementing RCTs lead to the exploration of alternative designs, such as pre-posttest studies and pragmatic trials. Pragmatic trials are designed to evaluate the effectiveness of interventions in real-life clinical settings, while benchmarking controlled trials (BCTs) aim to assess the efficacy of interventions or clinical pathways in observational, real-world settings. These designs are gaining popularity in PRM research due to their better alignment with the complexities of rehabilitation. Proper reporting of study details is essential for transparency and the replication of research [60,61,62].

The present scoping review does not examine the cost-effectiveness of the treatment options under investigation. It is advisable to consider the implementation of clinical practice guidelines that promote high-quality therapy within a well-equipped clinical setting, accompanied by adequate financial support. In accordance with the GRADE (grading of recommendations, assessment, development, and evaluation) strategy, we propose the adoption of high-quality treatments and their integration into clinical practice guidelines within an optimal clinical environment, accompanied by sufficient financial backing. This method has the potential to facilitate prompt and comprehensive recovery in individuals who have experienced a stroke, thereby enhancing their overall quality of life.

## 4. Conclusions

This review was carried out in the pattern of a modified scoping review in post-stroke spasticity. The aim of this review is to define the most recent and best evidence available for the management of spasticity to prevent complications in stroke patients. We highlight multimodality treatments for GRADE A evidence, which can be applied to improve functional recovery and quality of life.

## 5. Materials and Methods

### 5.1. Search Strategy and Selection Criteria

A scoping review of the latest articles that are high quality or “A” by GRADE [6,7,8] (i.e., systematic reviews, meta-analyses, and randomized control trials) regarding the treatments for post-stroke spasticity included in Figure 1 was performed according to the Preferred Reporting Items for Systematic Review and Meta-Analysis (PRISMA) guidelines [9] and the PRISMA Extension for Scoping Reviews (PRISMA-ScR) [10]. The data presented in Figure 1 encompass and consolidate information from previous studies. We selected this method to provide a thorough and comprehensive analysis of our findings with respect to the existing inquiry literature. In this section, we describe the specific criteria and standards we employed to assess and grade the evidence. The approach we utilized is an adapted iteration of the initial scoping review process. Furthermore, this study is an inaugural implementation of the modified scoping reviews by Areerat Suputtitada (AS), as it encompasses a wide range of treatments for post-stroke spasticity. We adhered to the PRISMA extension guidelines for conducting this scoping review (see the Supplementary file titled “Supplementary File S3-checklist”)

The quality of the evidence was based on the Grading of Recommendations, Assessment, Development, and Evaluation (GRADE) [6,7] approach, as shown in Table 2. There are various treatments for post-stroke spasticity that are published as GRADE A, making the available scoping review methodology able to be modified by AS. The corresponding flowchart is presented in Figure 1, and the included studies are presented in Table 1. Spasticity should be treated for multiple reasons, including the prevention of severe consequences and the improvement of mobility training. However, we believed that it would have been advantageous to differentiate between the two. This modified scoping review appropriately highlighted spasticity as the primary outcome rather than functional ability. There were specifics provided in the recommendations.

### 5.2. Modified Scoping Review by Areerat Suputtitada (AS)

This study used a modified scoping review from the original one [10] with the following details: All authors—three rehabilitation medicine physicians (AS, SC, and C.C.P.C) and one neurologist (D.M.S)—compiled a list of all treatments of interest for post-stroke spasticity, which totaled 32 interventions, from their diverse perspectives and considerations. Each of the 32 treatments was identified by searching a database. The packed bubble chart format, modified from the Evidence Alert System [11], as shown in Figure 2, which was employed for analyzing post-stroke spasticity interventions, utilizes two distinct visual methods:

1. Color coding: In this chart, various shades of blue were used to signify the quality of evidence for each type of intervention, with a spectrum ranging from dark navy to light blue. This gradation represented the quality of the evidence, categorized from GRADE A (highest quality, indicated by dark navy) to GRADE D (lower quality, denoted by light blue).
2. Circle size (circumference): The circumference of each circle within the chart also reflected the number of randomized controlled trials associated with each intervention. Larger circles indicated a higher number of trials. Smaller circles, on the other hand, represented a lower amount of evidence.

The treatments that did not receive an A GRADE were excluded. The published GRADE A treatments were identified by the randomized controlled trial, systematic review, and meta-analysis article types. The total number of publications for each of the selected GRADE A treatments was then identified through database searches. Then, each treatment selected as GRADE A was evaluated as GRADE A. Then, the eligibility of full-text articles was independently evaluated by two authors (AS and SC). Any differences were settled by a third author (C.C.P.C). The flow chart is presented in Figure 1. 

### 5.3. Eligibility Criteria

We included only English-language, grade “A” GRADE system studies on medical, rehabilitation, and surgical interventions for post-stroke spasticity published within the defined study period. Studies on spinal cord injury, cerebral palsy, or not meeting these criteria were excluded.

### 5.4. Information Sources

The focused interventions consisted of stretching exercises, static stretching with positional orthoses, constraint-induced movement therapy (CIMT), proprioceptive neuromuscular facilitation technique (PNF), heat modality, mirror therapy, sensory stimulation, hand splint, motor imagery, action observation therapy (AOT), intrathecal baclofen (ITB) in both severe cases and ambulatory cases, oral medication (i.e., diazepam, baclofen, eperisone, phenothiazine, and tizanidine), botulinum toxin A injection, diagnostic nerve block using anesthetic products, neurolysis, DREZotomy, rhizotomy, extracorporeal shock wave therapy (ESWT), transcutaneous electrical nerve stimulation (TENS), repetitive peripheral magnetic stimulation (rPMS), non-invasive brain stimulation (NIBS) (e.g., transcranial magnetic stimulation (TMS) and transcranial direct current stimulation (tDCS)), vibration therapy (e.g., whole-body vibration (WBV) and localized muscle vibration (LMV)), low-power laser therapy (LPL), and high-power laser therapy (HPL). A search of the literature was derived from research involving human subjects, published in English, and indexed in MEDLINE, PubMed, the Cochrane Library, and other selected databases relevant to this review published between 1 January 2000 and 31 August 2023.

### 5.5. Search and Selection of Sources of Evidence

A MeSH search was performed for each intervention using the PubMed Medline database with the following terms: intervention (i.e., stretching exercise/acupuncture) AND stroke AND spasticity; filters: randomized controlled trial, systematic review, and meta-analysis between 1 January 2000 and 31 August 2023, as noted in the Supplementary file titled “Supplementary File S1-PRISMA,” Relevant papers that were cited within the retrieved articles were also included. Two authors (AS and SC) independently performed the literature search and assessed the results before the synthesis. Any disagreement was resolved by a third author (C.C.P.C).

### 5.6. Risk of Bias 

We assessed the methodological quality using the GRADE approach; only GRADE A interventions were selected.

### 5.7. Data Charging Process and Data Items

A data-charting form was jointly developed by two reviewers (AS and SC) to determine which variables to extract. The two reviewers independently charted the data, discussed the results, and continuously updated the data-charting form in an iterative process. We abstracted the data on the type of study (systematic review/meta-analysis/randomized controlled trial), the number of trials included, the sample size of the participants, the characteristics of the participants (the population), the intervention, parameters, control (i.e., sham, placebo), outcome (i.e., Modified Ashworth Scale (MAS) and Ashworth Scale (AS)), and limitations. 

We have recorded all of the aspects of our search and review process. These records are included in the Supplementary file titled “Supplementary File S1-PRISMA”, which contains the detailed Medline search strategy for each intervention examined in our study. This comprehensive documentation ensures the transparency and replicability of our search methodology. Additionally, we have preserved the complete record of our search results. These records are securely stored on our digital drive and are readily available upon request. This step ensures that any interested parties can access our full search data for further inspection or verification.

## Figures and Tables

**Table 1:** Table of studies.

Authors	Types of Studies	RCTs/ Population	Population	Interventions	Parameters	Control	Outcome Measures	Future Studies/ Limitation
1. Stretching exercise								
Gomez et al., 2021 [13]	SR and meta- analysis	8 RCTs included in the SR; 6 RCTs included in the meta- analysis (332 patients)	Chronic stroke (3–6 months)	Passive static/ dynamic stretching by PT, self-stretching	Varied greatly, but none of them exceed 60 min/ session	No treatment	MAS *p* = 0.45, I^2^ = 92%	Larger sample size and optimal protocol
2. Static stretching with positioning orthoses								
Salazar et al., 2018 [14]	SR and meta- analysis	3 RCTs for spasticity outcome (57 patients)	Chronic stroke	Static stretching with wrist devices	6–7 days/week, 20–45 min/day (separated by 2–3 time/day), 3–4 weeks	No treatment	MAS *p* < 0.01, I^2^ = 82%	Larger sample size
3. Transcutaneous electrical nerve stimulation (TENS)								
Mahmood et al., 2018 [15]	SR and meta- analysis	15 RCTs included in the SR; 7 RCTs Included in the meta- analysis (427 patients)	Chronic stroke	TENS	High frequency (100 Hz), duration > 30 min/session, electrodes placement along the nerve/ muscle belly, intensity twice of the sensory threshold, and treatment duration > 2 weeks	No treatment, or placebo- controlled interventions, or active controls, or PT	MAS *p* = 0.001, I^2^ = 17%	Focusing on effect in UE, duration of efficacy, and effect of low TENS
4. Extracorporeal shock wave therapy (ESWT)								
Zhang et al., 2022 [16]	SR and meta- analysis	42 RCTs Included in the SR; 34 RCTs Included in the meta-analysis (1973 patients)	Upper motor neuron injury (29 studies focused on stroke)	ESWT	Radial ESWT >2 sessions	Sham ESWT/ conventional RT	MAS *p* < 0.0001, I^2^ = 78%	Focusing on the duration of efficacy, dose response relationship, mechanism
5. Repetitive peripheral magnetic stimulation (rPMS)								
Pan et al., 2022 [17]	SR and meta- analysis	8 RCTs included in the SR; 6 RCTs Included in the meta-analysis (297 patients)	Spastic paralysis patients (170 chronic stroke)	rPMS	5/25 Hz of frequency, 3–30 min/ session, and round/ figure of eight coil types	Sham rPMS and/or PT, and PT	AS, MAS, MTS, and FMA	Focusing on the duration of efficacy, with larger sample size, and optimal protocol
6. Non-invasive brain stimulation (NIBS)								
Wang et al., 2022 [18]	SR and meta- analysis	14 RCTs (232 patients on TMS, and 345 patients on tDCS)	Stroke patients	11 RCTs on TMS	Low frequency at unaffected hemisphere	Sham TMS, Sham TMS plus PT, Sham TMS plus RT	MAS in UE *p* < 0.00001, I^2^ = 3%	Further study in LE spasticity, mechanism, and the duration of efficacy
				7 RCTs on tDCS	Anodal stimulation at affected hemisphere, 0.7 mA or 1.2 mA	Sham tDCS, Sham tDCS plus PT, Sham tDCS plus VR	MAS in UE *p* = 0.003, I^2^ = 78%	
7. Botulinum toxin A (BoNT-A) injection								
7.1 In the upper and lower extremities								
Schnitzler et al., 2022 [19]	SR	49 primary studies	Spasticity of any Etiology (38 studies in stroke/ brain injury)	BoNT-A injection	AboBoNT-A	Placebo/ control or another BoNT-A treatment	AboBoNT-A dose given per muscle in clinical practice varies considerably, with only a slight trend toward a relationship between dose and muscle volume	-
7.2 In the upper extremities								
Sun et al., 2019 [20]	meta- analysis	27 RCTs (2793 patients) (16 trials UE; 12 trials of muscle tone in UE)	Stroke patients	BoNT- A injection	AboBoNT-A/ OnaBoNT-A/ IncoBoNT-A	Placebo	Muscle tone *p* < 0.001, I^2^ = 52.1%	-
Andringa et al., 2019 [21]	SR and meta- analysis	40 RCTs (2718 patients)	Stroke patients	UE BoNT-A injection		Placebo and/or PT	MAS and AS	-
7.3 In the lower extremities								
Doan et al., 2021 [22]	SR and meta- analysis	12 RCTs included in SR, 9 RCTs included in Meta- Analysis (1601patients)	PSS in LE	BoNT-A Injection in LE	300 units of OnaBoNT-A and 1000 units of AboBoNT-A	Placebo/ dose- ranging	MAS, AS at week 4th, 8th, and 12th	Further studies on functional improvement
8. Dry needling (DN)								
Fernández et al., 2021 [23]	SR and meta- analysis	7 RCTs (83 patients)	Stroke patients	DN	-	RT/ sham DN/ non-TrP DN	MAS, MMAS *p* = 0.0007, I^2^ = 66%: UE *p* = 0.18, LE *p* < 0.0001	Larger sample size and examining the long-term effect
9. Intrathecal baclofen (ITB)								
Creamer et al., 2018 [24,25]	Multi- center phase 4 RCT	60 patients (ITB: 31; control: 29)	PSS in ≥2 extremities and ASS of ≥3 in ≥2 affected LE	ITB	-	CMM with oral antispastic drugs	ASS in LE, NRS, and quality of life	Larger sample sizes and longer follow-up
10. Whole-body vibration (WBV)								
Zhang et al., 2023 [26]	SR and meta- analysis	11 RCTs (475 patients)	Stroke patients	WBV or PT with WBV	Variable	Sham WBV or PT	MAS	Focusing on the duration of efficacy, severe spasticity
11. Localized muscle vibration (LMV)								
Avvantaggiato et al., 2021 [27]	SR and meta- analysis	14 RCTs (425 patients)	Stroke patients	LMV plus PT	Variable from frequency 30, 80, 90, 91, 100, 120, and 300 Hz; amplitude 0.01, 0.2–0.5, 1, and 2 mm; duration 5, 20, 30, and 60 min	PT/sham plus PT	Neurophysiological parameters (TMS, ENG, and EMG), MAS (elbow: *p* = 0.001, I^2^ = 0%; wrist: *p* = 0.04, I^2^ = 36%; shoulder: *p* = 0.26, I^2^ = 0%) Functional scales	Larger size of homogeneous patients (shared methodology), on LE

SR: systematic review; RCTs: randomized controlled trials; PT: physical therapy; MAS: Modified Ashworth Scale; TENS: transcutaneous electrical nerve stimulation; ESWT: extracorporeal shock wave therapy; rPMS: repetitive peripheral magnetic stimulation; AS: Ashworth Scale; MTS: Modified Tardieu Scale; FMA: Fugl–Meyer Assessment; NIBS: non-invasive brain stimulation; TMS: transcranial magnetic stimulation; tDCS: transcranial direct current stimulation; UE: upper extremities; LE: lower extremities; BoNT-A inj.: botulinum toxin A injection; TrP: triggered point; DN: dry needling; MMAS: Modified Modified Ashworth Scale; RT: rehabilitation therapy; MA: manual acupuncture; ASS: Ashworth Scale Score; ITB: intrathecal baclofen; CMM: conventional medical management; NRS: numeric rating scale; WBV: whole-body vibration; LMV: localized muscle vibration, ENG: electroneurography; and EMG: electromyography.

**Table 2:** The quality of the evidence was based on the grading quality of the evidence and the strength of the recommendations in clinical practice guidelines [7]. RCTs = randomized controlled trials.

Rank	Explanation	Examples
**High**	Further research is very unlikely to change our confidence in the estimate of the effect	Randomized trials without serious limitations Well-performed observational studies with very large effects (or other qualifying factors)
**Moderate**	Further research is likely to have an important impact on our confidence in the estimate of effect and may change the estimate	Randomized trials with serious limitations Well-performed observational studies yielding large effects
**Low**	Further research is very likely to have an important impact on our confidence in the estimate of the effect and is likely to change the estimate	Randomized trials with very serious limitations Observational studies without special strengths or important limitations
**Very low**	Any estimate of the effect is very uncertain	Randomized trials with very serious limitations and inconsistent results Observational studies with serious limitations Unsystematic clinical observations (i.e., case series or case reports)

## Data Availability

The data presented in this study are available in this article or Supplementary Materials.

## Supplementary Materials

The following supporting information can be downloaded at: https://www.mdpi.com/article/10.3390/toxins16020098/s1, Supplementary File S1-PRISMA, Supplementary File S2-Risk of Bias, Supplementary File S3-checklist.
