# Supplementary material for: Best Practice Guidelines for the Management of Patients with Post-Stroke Spasticity: A Modified Scoping Review
Source: Toxins (Basel). 2024 Feb 10;16(2):98. doi: 10.3390/toxins16020098 (PMC10892074; doi:10.3390/toxins16020098)
Supplement: Supplementary file 1 [file toxins-16-00098-s001.zip › Supplementary File S2-Risk of bia.pdf]

Supplementary file: Risk of bias assessment

1. **Stretching** (PEDro 5.5 moderate/good methodological quality (range 4-8))

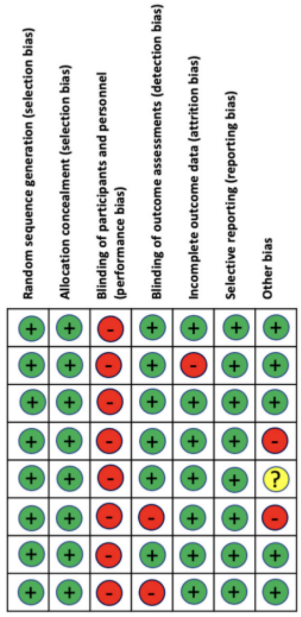

Study	Random sequence generation (selection bias)	Allocation concealment (selection bias)	Blinding of participants and personnel (performance bias)	Blinding of outcome assessments (detection bias)	Incomplete outcome data (attrition bias)	Selective reporting (reporting bias)	Other bias
Pradhan et Bansal (2018)	+	+	-	+	+	+	+
Ghasemi et al. (2018)	+	+	-	+	-	+	+
Ghasemi et al. (2018)	+	+	-	+	+	+	+
Jang et al. (2016)	+	+	-	+	+	+	-
Santamato et al. (2015)	+	+	-	+	+	+	?
Kim et al. (2013)	+	+	-	-	+	+	-
Carda et al. (2011)	+	+	-	+	+	+	+
Jung et al. (2011)	+	+	-	-	+	+	+

Figure 2. Risk of bias of the studies included in the systematic review.

Reference: Gomez-Cuaresma L, Lucena-Anton D, Gonzalez-Medina G, Martin-Vega FJ, Galan-Mercant A, Luque-Moreno C. Effectiveness of Stretching in Post-Stroke Spasticity and Range of Motion: Systematic Review and Meta-Analysis. J Pers Med. 2021;11(11).

Authors' perspectives: Stretching intervention could not perform participants and personnel blinding (performance bias). The risk of bias of this systematic review is acceptable.

2. **Static stretching with positioning orthoses** (median PEDro = 6 (range 4-9))

Table 2
PEDro scores of included studies.

Study	Random allocation	Concealed allocation	Groups similar at baseline	Participant blinding	Therapist blinding	Assessor blinding	Adequate follow-up	Intention-to-treat analysis	Between-group difference reported	Point estimate and variability reported	Total score (0 to 10)
Ada et al., 2005	✓		✓			✓		✓			4
DeJong et al., 2006	✓	✓	✓			✓	✓		✓	✓	7
De Jong et al., 2013	✓	✓	✓	✓		✓	✓	✓	✓	✓	9
Dean et al., 2000	✓	✓	✓			✓		✓		✓	6
Gustafsson et al., 2006	✓	✓	✓	✓		✓				✓	6
Horsley et al., 2007	✓	✓	✓			✓	✓	✓	✓	✓	7
Jang et al., 2016	✓		✓			✓				✓	4
Jung et al., 2011	✓		✓						✓	✓	4
Kim et al., 2013	✓		✓						✓	✓	4
Lanin et al., 2007	✓	✓	✓			✓	✓	✓		✓	7
Turton et al., 2005	✓	✓	✓			✓				✓	6
										Median (range)	6 (4–9)

Reference: Salazar AP, Pinto C, Ruschel Mossi JV, Figueiro B, Lukrafka JL, Pagnussat AS. Effectiveness of static stretching positioning on post-stroke upper-limb spasticity and mobility: Systematic review with meta-analysis. Ann Phys Rehabil Med. 2019;62(4):274-82.

Authors' perspectives: Static stretching with positioning orthoses actually could not perform participants and personnel blinding (performance bias). The risk of bias of this systematic review is acceptable.

3. Transcutaneous Electrical Nerve Stimulation (TENS)

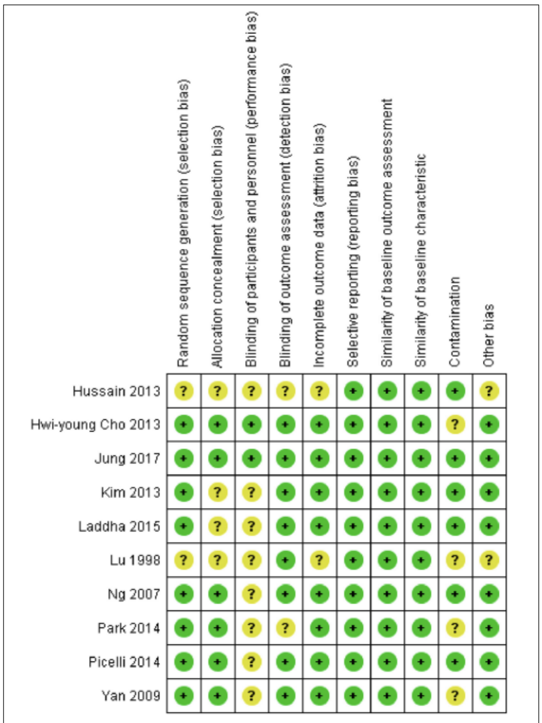

	Random sequence generation (selection bias)	Allocation concealment (selection bias)	Blinding of participants and personnel (performance bias)	Blinding of outcome assessment (detection bias)	Incomplete outcome data (attrition bias)	Selective reporting (reporting bias)	Similarity of baseline outcome assessment	Similarity of baseline characteristic	Contamination	Other bias
Hussain 2013	?	?	?	?	?	+	+	+	+	?
Hwi-young Cho 2013	+	+	+	+	+	+	+	+	?	+
Jung 2017	+	+	+	+	+	+	+	+	+	+
Kim 2013	+	?	?	+	+	+	+	+	+	+
Laddha 2015	+	?	?	+	+	+	+	+	+	+
Lu 1998	?	?	?	+	?	+	+	+	?	?
Ng 2007	+	+	?	+	+	+	+	+	+	+
Park 2014	+	+	?	?	+	+	+	+	?	+
Picelli 2014	+	+	?	+	+	+	+	+	+	+
Yan 2009	+	+	?	+	+	+	+	+	?	+

Fig 2 Risk of bias summary for the included RCTs.

Reference: Mahmood A, Veluswamy SK, Hombali A, Mullick A, N M, Solomon JM. Effect of Transcutaneous Electrical Nerve Stimulation on Spasticity in Adults With Stroke: A Systematic Review and Meta-analysis. Arch Phys Med Rehabil. 2019;100(4):751-68.

Authors' perspectives: Transcutaneous Electrical Nerve Stimulation could not perform participants and personnel blinding (performance bias). The risk of bias of this systematic review is acceptable.

4. Extracorporeal shock wave therapy (ESWT)

TABLE 1. ROB 2.0 assessment results of the included studies

Study	Domain 1	Domain 2	Domain 3	Domain 4	Domain 5	Overall
S1[28]	Some	Low	Low	Low	Some	Some
S2[41]	Some	High	High	Low	Some	High
S3[42]	Some	Some	Low	High	Some	High
S4[43]	Some	High	High	Low	Some	High
S5[44]	Some	Some	Low	Low	Some	Some
S6[45]	Some	Some	Low	Low	Some	Some
S7[46]	Low	Some	Low	Low	Some	Some
S8[47]	Some	Some	Low	Low	Some	Some
S9[48]	Some	Low	Low	Low	High	High
S10[49]	Some	Some	Low	High	Some	High
S11[50]	Some	Some	Low	Low	Some	Some
S12[51]	Some	High	High	High	Some	High
S13[52]	Some	High	High	High	Some	High
S14[53]	Some	High	High	High	Some	High
S15[54]	Low	High	High	High	Some	High
S16[55]	Low	Some	Low	Low	Some	Some
S17[56]	Some	Some	Low	Low	Some	Some
S18[57]	Some	Some	Low	Low	Some	Some
S19[58]	Some	Some	Low	Low	Some	Some
S20[59]	Some	Some	Low	High	Some	High
S21[60]	Some	Some	Low	High	Some	High
S22[61]	Some	Some	Low	High	Some	High
S23[62]	Low	Some	Low	Low	Some	Some
S24[63]	Some	Some	Low	Low	Some	Some
S25[64]	Some	Some	Low	Low	Some	Some
S26[65]	Some	Some	Low	High	Some	High
S27[66]	Some	Some	Low	Low	Some	Some
S28[67]	Some	High	High	Low	Some	High
S29[68]	Low	Some	Low	Low	Some	Some
S30[69]	Some	Some	Low	High	Some	High
S31[70]	Some	Low	Low	Low	Some	Some
S32[71]	Some	Some	Low	Low	Some	Some
S33[72]	Some	Some	Low	High	Some	High
S34[73]	Some	Some	Low	High	Some	High
S35[74]	Some	Some	Low	Low	Some	Some
S36[75]	Some	Some	Low	High	Some	High
S37[76]	Some	Some	Low	High	Some	High
S38[77]	Some	Some	Low	High	Some	High
S39[78]	Some	Some	Low	High	Some	High
S40[79]	Some	Some	Low	Some	Some	Some
S41[80]	Low	Some	Low	Some	Some	Some
S42[81]	Some	Some	Low	High	Some	High

Domain 1, risk of bias arising from the randomization process; domain 2, risk of bias due to deviations from the intended interventions (effect of assignment to intervention); domain 3, risk of bias due to missing outcome data; domain 4, risk of bias in measurement of the outcome; domain 5, risk of bias in selection of the reported result; high, high risk of basis; low, low risk of basis; ROB 2.0, version 2 of the Cochrane risk-of-bias tool; S, study; some, some concerns.

Reference: Zhang HL, Jin RJ, Guan L, Zhong DL, Li YX, Liu XB, et al. Extracorporeal Shock Wave Therapy on Spasticity After Upper Motor Neuron Injury: A Systematic Review and Meta-analysis. Am J Phys Med Rehabil. 2022;101(7):615-23.

Authors' perspectives: From ROB 2.0, there are 13 out of 29 studies on poststroke spasticity, which are highlighted in yellow, that revealed a high risk of bias in overall domains. However, there is a high risk of bias only in domain 4 in 8 of the 13 studies. Domain 4 is the risk of bias in measurement of the outcome, in which all 8 studies measured the Modified Ashworth Scale, which is considered a subjective measurement. This means the importance of further research in objective measurements for decreasing the risk of bias in domain 4 when doing the study of spasticity interventions.

5. Repetitive peripheral magnetic stimulation (rPMS)

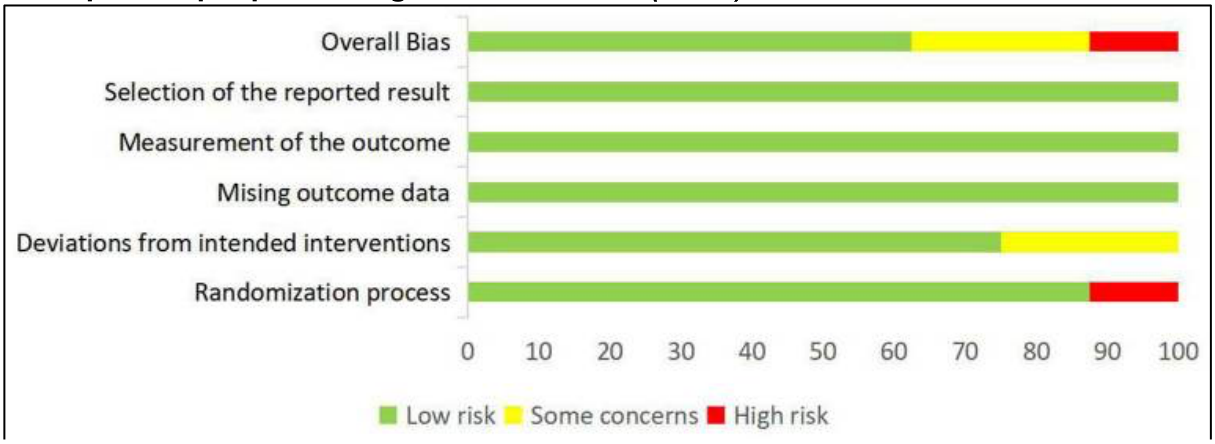

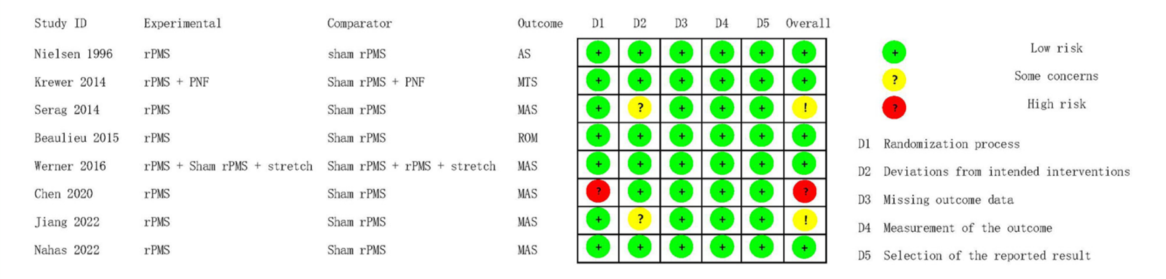

FIGURE 3
Risk of bias assessment of individual studies: no difference in the results for the outcomes assessed in each study.

Reference: Pan JX, Diao YX, Peng HY, Wang XZ, Liao LR, Wang MY, et al. Effects of repetitive peripheral magnetic stimulation on spasticity evaluated with modified Ashworth scale/Ashworth scale in patients with spastic paralysis: A systematic review and meta-analysis. Front Neurol. 2022;13:997913.

Authors' perspectives: The studies under review consistently demonstrated a low risk of bias in the selection of reported results, primarily because the outcome analyses aligned with the pre-defined analysis plan. Consequently, the majority of these studies are characterised by a low to moderate risk of bias.

6. Non-invasive brain stimulation (NIBS)

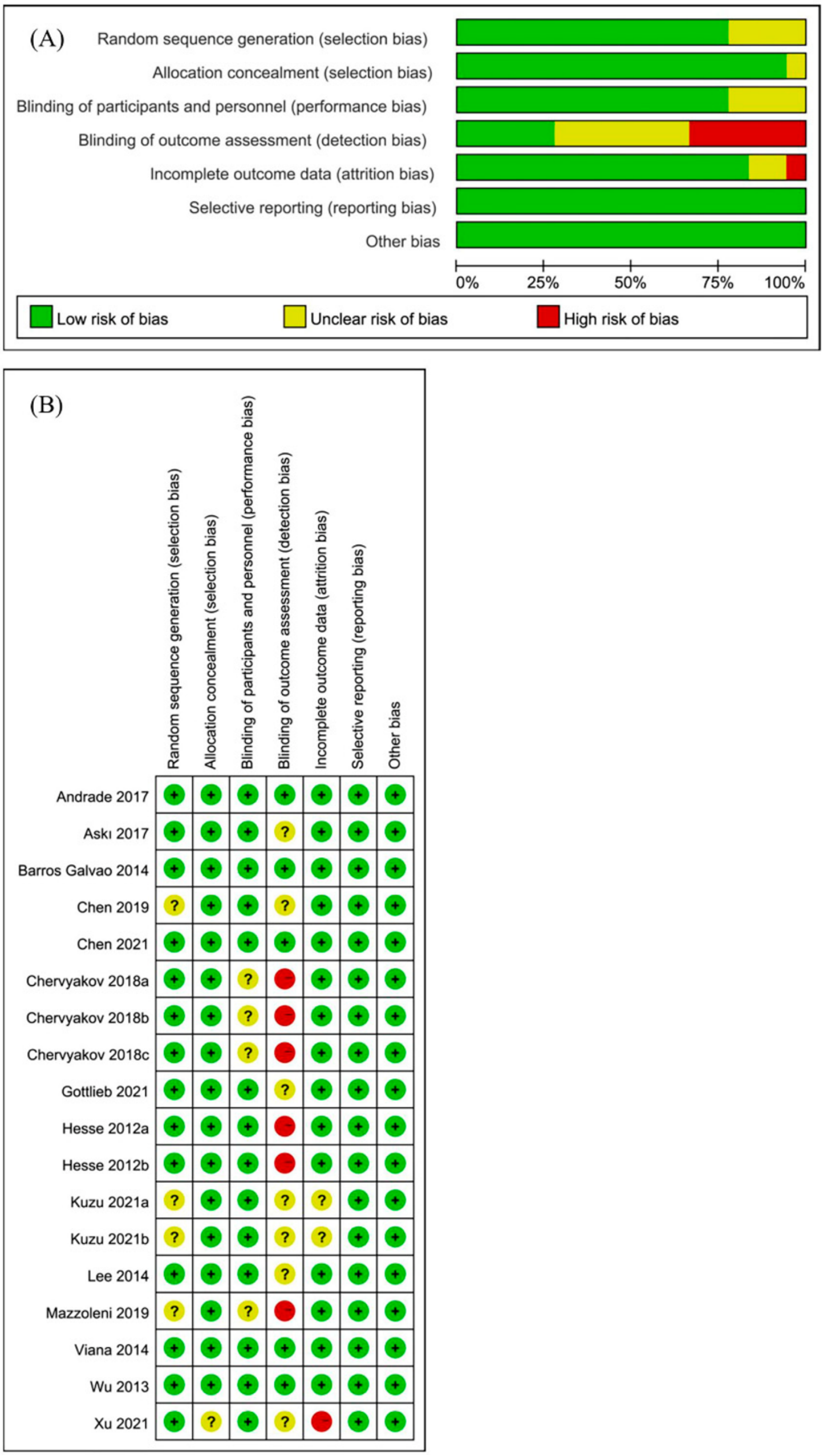

Reference: Wang X, Ge L, Hu H, Yan L, Li L. Effects of Non-Invasive Brain Stimulation on Post-Stroke Spasticity: A Systematic Review and Meta-Analysis of Randomized Controlled Trials. Brain Sci. 2022;12(7).

Authors' perspectives: Overall risk of bias is acceptable.

7. Botulinum toxin A (BoNT-A) injection

Table A1. Quality assessment of quantitative randomized controlled trials.

First Author, Year	Is Randomization Appropriately Performed?	Are the Groups Comparable at Baseline?	Are There Complete Outcome Data?	Are Outcome Assessors Blinded to the Intervention Provided?	Did the Participants Adhere to the Assigned Intervention?
Bakheit, 2000 [12]	Can't tell	Yes	Yes	Can't tell	Yes
Bakheit, 2001 [13]	Yes	Yes	Yes	Yes	Yes
Baricich, 2008 [17]	Yes	Yes	Yes	Can't tell	Yes
Bhakta, 2000 [12]	Yes	Yes	Yes	Yes	Yes
Burbaud, 1996 [21]	Can't tell	Yes	Yes	Can't tell	Yes
Gracies, 2017 [28]	Yes	Yes	Yes	Yes	Yes
Hesse, 1995 [32]	Can't tell	Yes	Can't tell	Can't tell	Yes
Hesse, 1998 [33]	Yes	Yes	Yes	Can't tell	Yes
Johnson, 2002 [35]	Yes	Yes	Yes	Can't tell	Yes
Kong, 2007 [36]	Yes	Yes	Yes	Yes	Yes
Lam, 2012 [37]	Yes	Yes	Yes	Yes	Yes
Marco, 2007 [39]	Yes	Yes	Yes	Yes	Yes
McCrory, 2009 [40]	Yes	Yes	Yes	Yes	Yes
O'Dell, 2018 [43]	Can't tell	Yes	Yes	Can't tell	Yes
Picelli, 2014 [47]	Yes	Yes	Yes	Yes	Yes
Picelli, 2016 [48]	Yes	Yes	Yes	Yes	Yes
Rekand, 2019 [50]	Yes	Yes	Yes	Yes	Yes
Rosales, 2012 [51]	Yes	Yes	Yes	Yes	Yes
Shaw, 2010 [52]	Yes	Yes	Yes	Yes	Yes
Sun, 2010 [53]	Yes	Yes	Yes	Yes	Yes

Download

Table A1. *Cont.*

First Author, Year	Is Randomization Appropriately Performed?	Are the Groups Comparable at Baseline?	Are There Complete Outcome Data?	Are Outcome Assessors Blinded to the Intervention Provided?	Did the Participants Adhere to the Assigned Intervention?
Suputtitada, 2005 [54]	Yes	Yes	Yes	Yes	Yes
Yazdchi, 2013 [57]	Yes	Can't tell	Yes	Can't tell	Can't tell
Yelnik, 2007 [58]	Yes	Yes	Yes	Yes	Yes

Table A2. Quality assessment of quantitative non-randomized studies.

First Author, Year	Are the Participants Representative of the Target Population?	Are Measurements Appropriate Regarding Both the Outcome and Intervention (or Exposure)?	Are There Complete Outcome Data?	Are the Confounders Accounted for in the Design and Analysis?	During the Study Period, Is the Intervention Administered (or Exposure Occurred) as Intended?
Bakheit, 2002 [14]	Can't tell	Yes	Yes	Can't tell	Yes
Barden, 2014 [16]	Yes	Yes	Yes	Can't tell	Yes
Carvalho, 2018 [23]	Yes	Yes	Yes	Can't tell	Yes
de Niet, 2015 [24]	No	Yes	Yes	Yes	Yes
Frasson, 2005 [26]	Yes	Yes	Yes	Can't tell	Yes
Ghroubi, 2020 [27]	Yes	Yes	Yes	Can't tell	Yes
Turner-Stokes, 2013 [55]	Yes	Yes	Yes	Yes	Yes

Reference: Schnitzler A, Dince C, Freitag A, Iheanacho I, Fahrbach K, Lavoie L, et al. AbobotulinumtoxinA Doses in Upper and Lower Limb Spasticity: A Systematic Literature Review. Toxins. 2022;14(11):734.

Table 2 Methodological quality of included studies (PEDro Scale)

Study	1	2	3	4	5	6	7	8	9	10	11	Total
Bakheit et al[32]	1	1	0	1	1	0	1	1	1	1	1	8
Bakheit et al[35]	1	1	1	1	1	0	1	1	1	1	1	9
Bhakta et al[33]	1	1	1	1	1	1	1	1	1	1	1	10
Bhakta et al[45]	1	1	1	1	1	1	1	1	1	1	1	10
De Boer et al[46]	1	1	0	1	1	0	0	1	0	1	1	6
Brashear et al[36]	1	1	0	1	1	1	1	1	0	1	1	8
Brashear et al[37]	1	1	1	1	1	1	1	1	0	1	1	9
Childers et al[38]	1	1	1	1	1	1	0	0	0	1	1	7
Cousins et al[52]	1	1	1	0	1	0	1	0	0	1	1	6
Elovic et al[65]	1	1	1	1	1	0	0	1	0	1	1	7
Gracies et al[62]	1	1	1	1	1	1	1	1	0	1	1	9
Gracies et al[22]	1	1	1	1	1	1	1	1	1	1	1	10
Guo et al[40]	1	1	0	1	0	0	0	1	0	1	1	5
Hesse et al[31]	1	1	0	1	1	0	1	0	0	1	1	6
Hesse et al[57]	1	1	1	1	0	0	1	1	0	1	1	7
Jahangir et al[41]	1	1	0	1	0	0	0	0	0	1	1	4
Kaji et al[53]	1	1	1	1	0	0	0	1	1	1	1	7
Kanovsky et al[48]	1	1	1	0	0	0	0	1	1	1	1	6
Kong et al[42]	1	1	1	1	1	1	1	1	0	1	1	9
Lim et al[47]	1	1	1	1	1	0	1	0	1	1	1	8
Marciniak et al[58]	1	1	1	1	1	1	1	1	1	1	1	10
Marciniak et al[66]	1	1	1	1	1	1	1	1	0	0	1	8
Marco et al[43]	1	1	1	1	1	1	1	1	0	1	1	9
McCrory et al[49]	1	1	1	1	1	1	1	1	1	1	1	10
Meythaler et al[50]	1	1	1	1	1	1	0	1	0	1	1	8
O'Dell et al[67]	1	1	1	1	1	1	0	0	0	0	1	6
Pennati et al[64]	1	1	1	0	0	0	1	1	0	0	1	5
Rosales et al[59]	1	1	1	1	1	1	1	1	1	1	1	10
Shaw et al[54,56]	1	1	1	1	0	0	1	1	1	1	1	8
Simpson et al[30]	1	1	0	1	1	1	0	1	0	1	1	7
Simpson et al[51]	1	1	0	1	1	1	1	1	1	1	1	9
Smith et al[34]	1	1	0	0	1	0	1	0	0	1	1	5
Suputtitada et al[39]	1	1	0	1	1	0	1	0	0	1	1	6
Turner-Stokes et al[56]	1	1	1	1	1	1	1	1	0	1	1	9
Umar et al[68]	1	1	1	1	1	0	0	1	1	1	1	8
Ward et al[63]	1	1	0	1	1	0	0	1	1	1	1	7
Wolf et al[60]	1	1	0	1	1	1	0	1	1	1	1	8
Yazdchi et al[61]	1	1	0	0	0	0	1	1	0	0	1	4
Yelnik et al[44]	1	1	1	1	0	0	0	1	0	1	1	6

NOTE. 0 indicates no; 1 indicates yes; and total score is the sum score item 2-11 (internal validity).

Reference: Andringa A, van de Port I, van Wegen E, Ket J, Meskers C, Kwakkel G. Effectiveness of Botulinum Toxin Treatment for Upper Limb Spasticity Poststroke Over Different ICF Domains: A Systematic Review and Meta-Analysis. Arch Phys Med Rehabil. 2019;100(9):1703-25.

Table S1. The quality of studies basing on the PEDro criteria.

Study	Specified eligibility criteria	Random allocation	Concealment of allocation	Comparable at baseline	Blinding of subjects	Blinding of therapist	Blinding of assessors	key outcome data for at least 85% of the subjects	Analysis by intention to treat	Between group analysis	Point estimate and variability	Total PEDro Score
Burbaud.1996	1	1	1	1	1	1	1	1	1	1	1	10
Pittock.2003	1	1	1	1	1	1	1	1	0	1	1	9
Mancini.2005	1	1	1	1	1	1	1	1	1	1	1	10
Kaji.2010	1	1	1	1	1	1	1	1	0	1	1	9
Dunne.2012	1	1	1	1	1	1	1	1	0	1	1	9
Pimentel.2014	1	1	1	1	1	1	1	1	0	1	1	9
Fietzek.2014	1	1	1	1	1	1	1	1	0	1	1	9
Tao.2015	1	1	1	1	1	1	1	1	1	1	1	10
Jiang Li. 2017	1	1	1	1	1	1	1	1	1	1	1	10
Gracies.2017	1	1	1	1	1	1	1	1	0	1	1	9
Wein.2018	1	1	1	1	1	1	1	1	0	1	1	9
Kerzoncuf. 2019	1	1	1	1	1	1	1	1	0	1	1	9

Reference: Doan TN, Kuo MY, Chou LW. Efficacy and Optimal Dose of Botulinum Toxin A in Post-Stroke Lower Extremity Spasticity: A Systematic Review and Meta-Analysis. Toxins (Basel). 2021;13(6).

3.2. Study Characteristics. Of the 27 included trials, 18 were conducted in patients with upper limb spasticity [27–44], and the remaining 9 studies in individuals with lower limb spasticity [45–53]. Studies were published from 1996 to 2018, with 20-468 patients included in each trial. Twenty of the included trials were conducted in Western countries, 6 in Eastern countries, and the remaining 1 in multiple countries [53]. The mean patient age ranged from 49.3 to 63.5 years, with a percentage of males ranging from 40.0 to 80.0. Study quality was evaluated using the JADAD scale; 10 trials had a score of 5, 8 had 4 points, 8 had 3 points, and the remaining 1 had 2 points.

Muscle tone

study	smd	smdl	smdu	publication year	mean age	percentage male	time since event	study quality
Simpson 1996	0.07	-0.83	0.97	<2010	>55	<60	>24	low
Hesse 1998	-0.5	-1.65	0.65	<2010	<55	>60	<24	low
Brashear 2002	-0.66	-1.02	-0.3	<2010	>55	<60	>24	high
Childers 2004	-1.01	-1.62	-0.39	<2010	>55	>60	>24	high
Simpson 2009	-1.05	-1.72	-0.38	<2010	<55	<60	<24	high
Meythaler 2009	-0.27	-1.14	0.59	<2010	<55	>60	<24	high
Mccrory 2009	-1.11	-1.55	-0.66	<2010	>55	>60	>24	high
Kaji 2010	-0.68	-1.18	-0.18	>2010	>55	>60	>24	high
Rosales 2012	-1.31	-1.65	-0.97	>2010	>55	>60	<24	high
Gracies 2015	-0.74	-1.07	-0.41	>2010	<55	>60	>24	high
Elovic 2016	-0.52	-0.78	-0.26	>2010	>55	<60	>24	high
Prazeres 2018	-0.36	-1.22	0.51	>2010	<55	>60	>24	high

Muscle tone

study	smd	smdl	smdu	publication year	mean age	percentage male	time since event	study quality
Burbaud 1996	-0.21	-1.03	0.62	<2010	<55	>60	>24	low
Kaji 2010	0.25	-0.11	0.61	>2010	<55	>60	<24	high
Fietzek 2014	0.72	0.16	1.28	>2010	<55	<60	<24	high
Ding 2015	0.02	-0.46	0.49	>2010	>55	<60	<24	low
Ding 2017	-1.4	-1.89	-0.91	>2010	>55	<60	<24	low

Reference: Sun, L.-C.; Chen, R.; Fu, C.; Chen, Y.; Wu, Q.; Chen, R.; Lin, X.; Luo, S. Efficacy and Safety of Botulinum Toxin Type A for Limb Spasticity after Stroke: A Meta-Analysis of Randomized Controlled Trials. BioMed Res. Int. 2019, 2019, 8329306.

Authors' perspectives: Overall risk of bias of all systematic review and meta-analysis are acceptable.

8. Dry needling (DN)

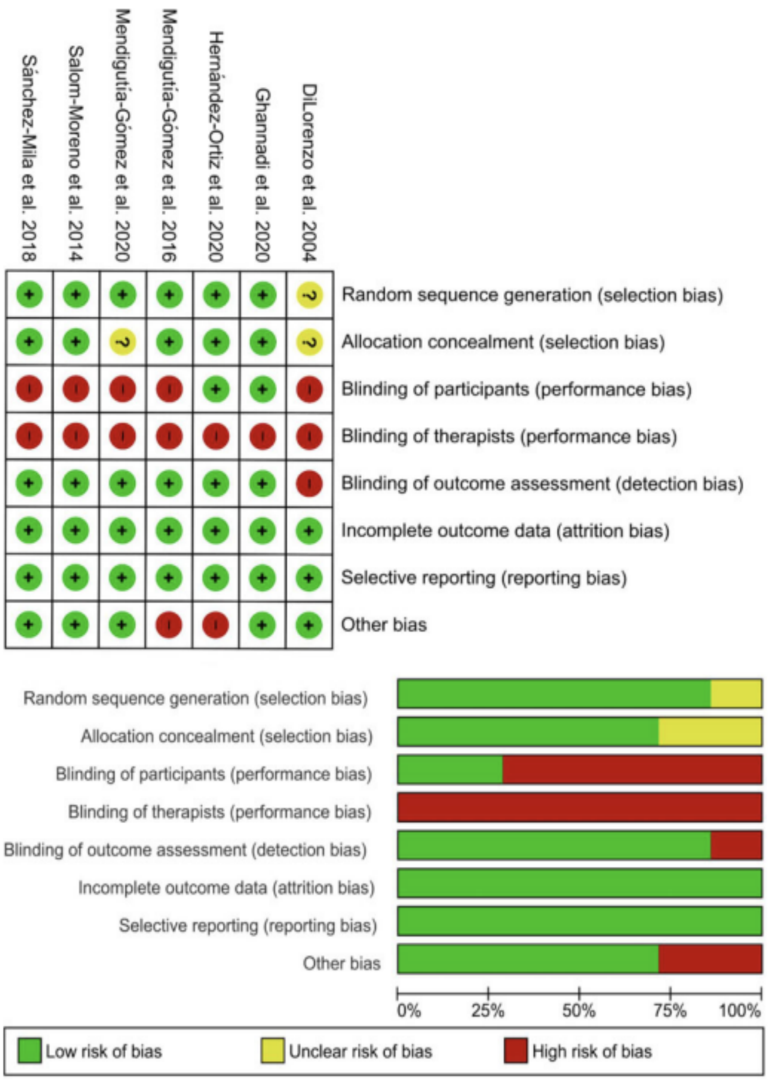

Table 2. Score of randomized clinical trials with PEDro scale

	1	2	3	4	5	6	7	8	9	10	Total
Dry needling in the lower extremity											
Salom-Moreno, et al. (2014) [28]	Y	Y	Y	N	N	Y	Y	N	Y	Y	7/10
Sánchez-Mila, et al. (2018) [29]	Y	Y	Y	N	N	Y	Y	N	Y	Y	7/10
Ghannadi, et al. (2020) [30]	Y	Y	Y	Y	N	Y	Y	N	Y	Y	8/10
Dry needling in the upper extremity											
DiLorenzo, et al. (2004) [31]	Y	N	Y	N	N	N	Y	N	Y	Y	4/10
Mendigutía-Gómez, et al. (2016) [32]	Y	Y	Y	N	N	Y	Y	N	Y	Y	7/10
Hernández-Ortiz, et al. (2020) [34]	Y	Y	Y	Y	N	Y	Y	Y	Y	Y	9/10
Mendigutía, et al. (2020) [33]	Y	Y	Y	N	N	Y	Y	Y	Y	Y	8/10

1: Random allocation of participants; 2: concealed allocation; 3: similarity between groups at baseline; 4: participant blinding; 5: therapist blinding; 6: assessor blinding; 7: fewer than 15% dropouts; 8: intention-to-treat analysis; 9: between-group statistical comparisons; 10: point measures and variability data.

Reference: Fernández-de-Las-Peñas C, Pérez-Bellmunt A, Llurda-Almuzara L, Plaza-Manzano G, De-la-Llave-Rincón AI, Navarro-Santana MJ. Is Dry Needling Effective for the Management of Spasticity, Pain, and Motor Function in Post-Stroke Patients? A Systematic Review and Meta-Analysis. Pain Med. 2021;22(1):131-41.

Authors' perspectives: Dry needling could not perform blinding of participants (performance bias) and therapist blinding (performance bias). The risk of bias of this systematic review is acceptable.

9. **Intrathecal baclofen (ITB)**
Author's perspective: PEDro = 7/10 (Eligibility criteria: +; Random allocation: Yes; Concealed allocation: Yes; Baseline comparability: Yes; Blind subjects: No; Blind therapists: No; Blind assessors: Yes; Adequate follow-up: No; Intention-to-treat analysis: yes; Between-group comparisons: Yes; Point estimates and variability: Yes). ITB could not perform blinding of participants and therapist blinding. The risk of bias of this systematic review is acceptable.

10. **Whole-body vibration therapy (WBV)**

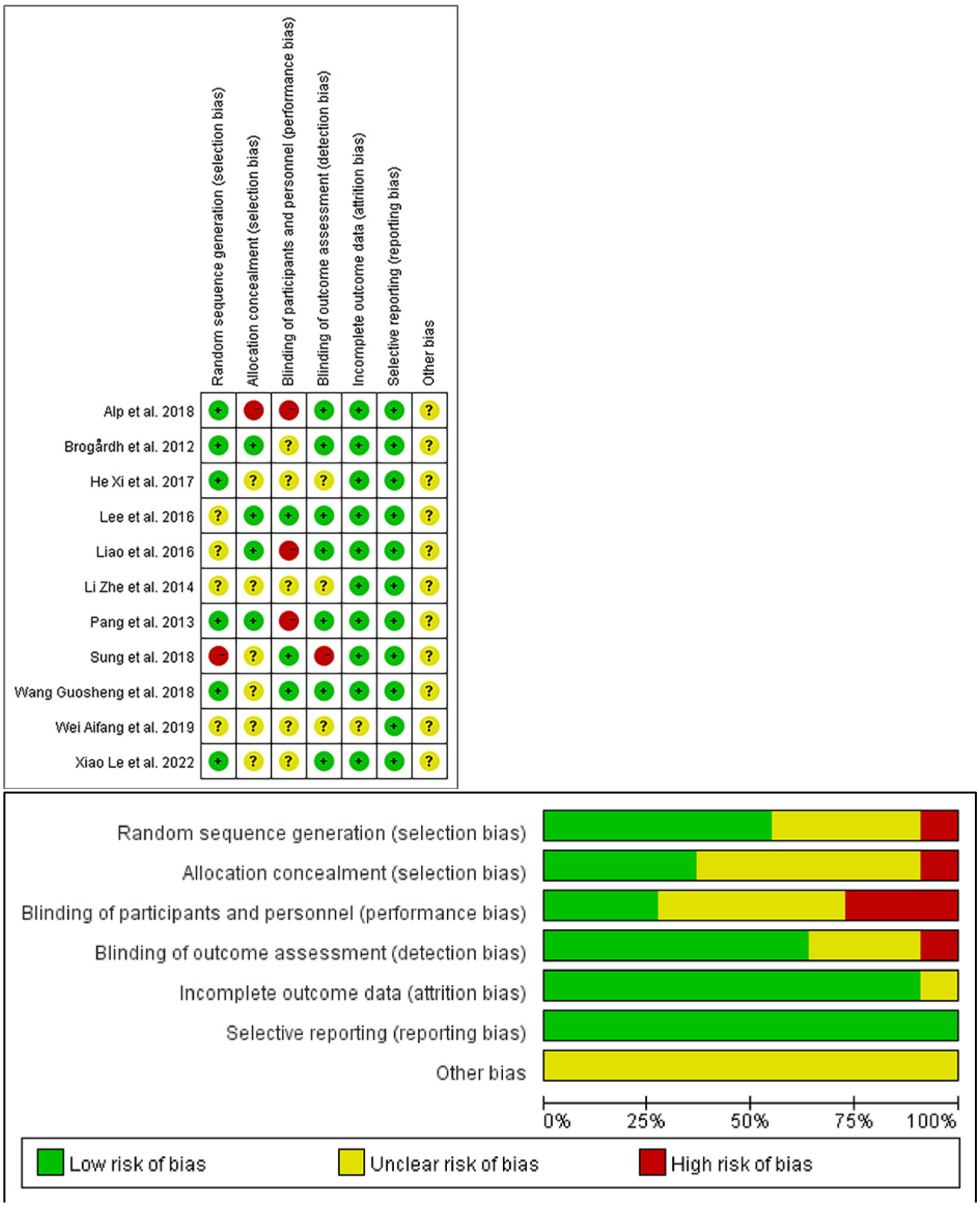

Reference: Zhang Q, Zheng S, Li S, Zeng Y, Chen L, Li G, et al. Efficacy and safety of whole-body vibration therapy for post-stroke spasticity: A systematic review and meta-analysis. Front Neurol. 2023;14:1074922.

Authors' perspectives: WBV could not perform blinding of participants (performance bias) and therapist blinding (performance bias). The risk of bias of this systematic review is acceptable.

11. Localized muscle vibration (LMV)

TABLE II.—*Methodology quality.*

Author	Random sequence generation (selection bias)	Allocation concealment (selection bias)	Blinding of participants and personnel (performance bias)	Blinding of outcome assessment (detection bias)	Incomplete outcome data (attrition bias)	Selective reporting (reporting bias)	Other bias
Annino *et al.*[25]	+	§	§	+	+	+	+
Calabrò *et al.*[26]	+	?	+	+	+	+	+
Casale *et al.*[27]	+	?	+	+	+	+	+
Costantino *et al.*[28]	+	?	+/ §	+/?	+	+	+
Caliandro *et al.*[29]	+	+	?	+	+	+	?
Celletti *et al.*[30]	+	+	+	+	?	?	+
Marconi *et al.*[31]	+	?	+	+	+	+	?
Noma *et al.*[32]	?	?	§	§	+	§	+
Paoloni 2014[33]	+	+	§	+	+	+	+
Paoloni 2010[34]	+	+	§	+	+	+	+
Tavernese *et al.*[35]	+	+	§	+	+	+	+
Toscano *et al.*[36]	+	?	+	+	+	+	+
Choi[37]	?	?	?	?	+	+	?
Lee *et al.*[38]	+	+	+	+	?	+	+

+: low risk; ?: uncertain risk; §: high risk; *functional tests and VNRS/MAS and clinical scales.

Reference: Avvantaggiato C, Casale R, Cinone N, Facciorusso S, Turitto A, Stuppiello L, et al. Localized muscle vibration in the treatment of motor impairment and spasticity in post-stroke patients: a systematic review. Eur J Phys Rehabil Med. 2021;57(1):44-60.

Authors' perspectives: LMV could not perform blinding of participants (performance bias) and therapist blinding (performance bias). The risk of bias of this systematic review is acceptable.
