# Supplementary material for: Best Practice Guidelines for the Management of Patients with Post-Stroke Spasticity: A Modified Scoping Review
Source: Toxins (Basel). 2024 Feb 10;16(2):98. doi: 10.3390/toxins16020098 (PMC10892074; doi:10.3390/toxins16020098)

Medline Search Strategy (Literature Search performed: January 1 2000- Aug 31, 2023)

Appendix fig. 1: Stretching AND spasticity AND stroke

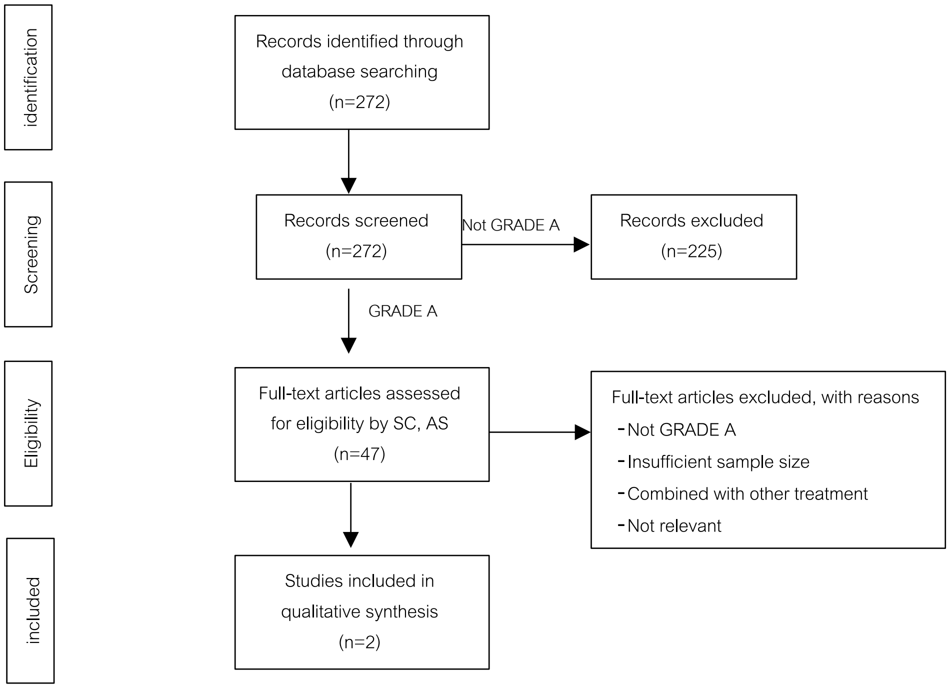

Appendix fig. 2: TENS AND spasticity AND stroke

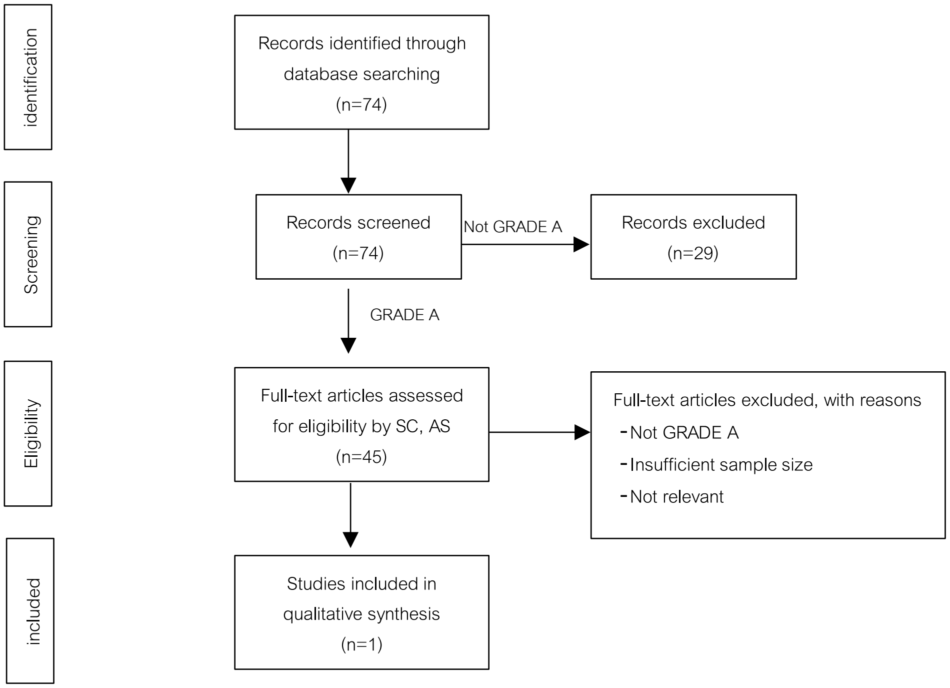

Appendix fig. 3: Shock wave AND spasticity AND stroke

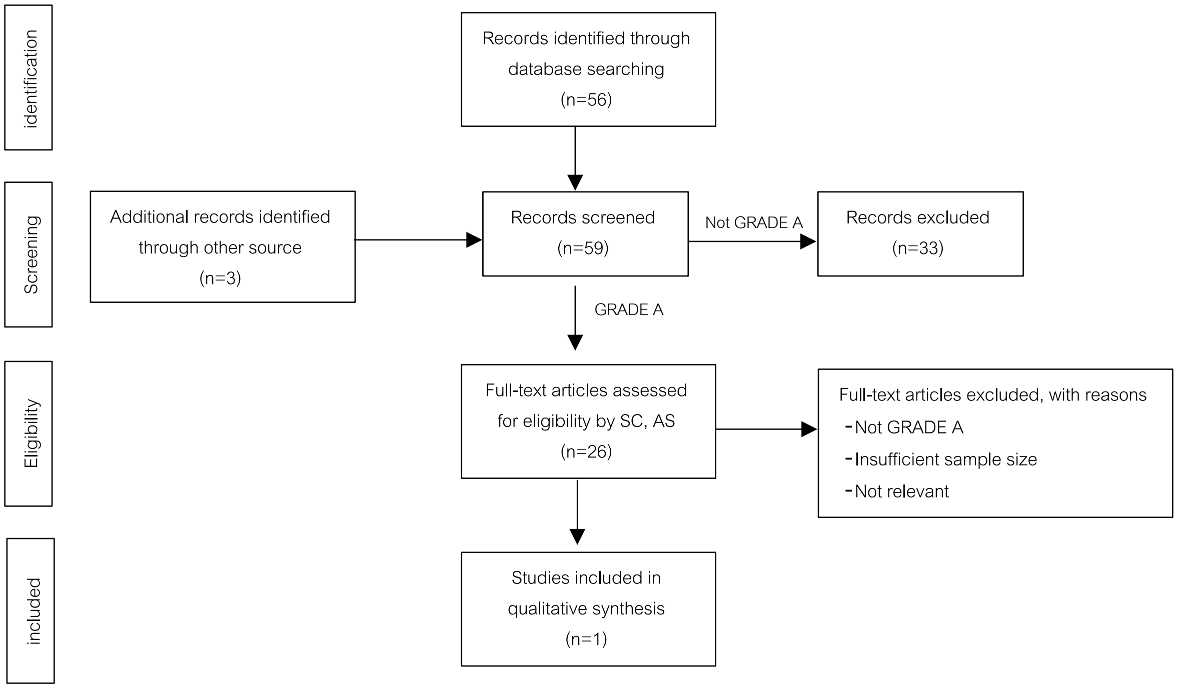

Appendix fig. 4: Peripheral Magnetic Stimulation AND spasticity AND stroke

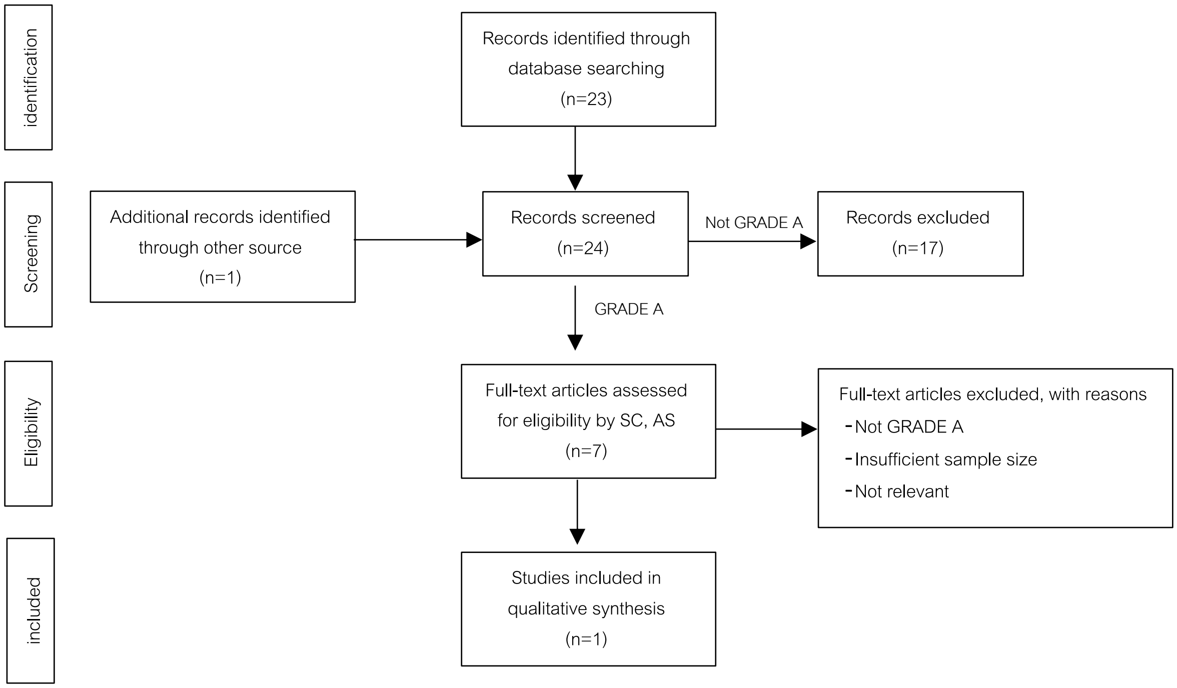

Appendix fig. 5: Transcranial Magnetic Stimulation AND spasticity AND stroke

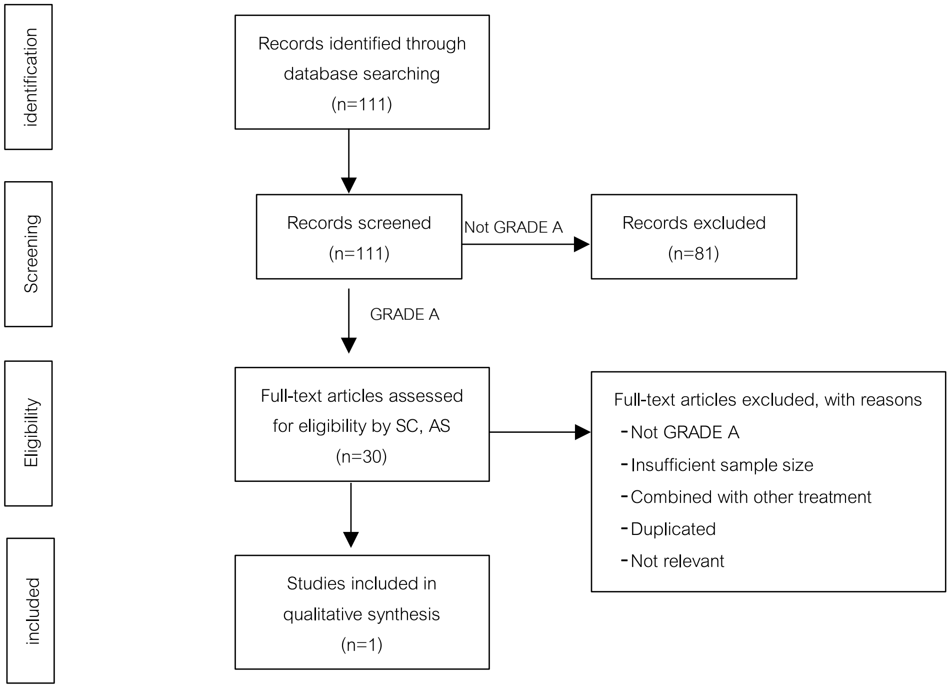

Appendix fig. 6: transcranial Direct Current Stimulation AND spasticity AND stroke

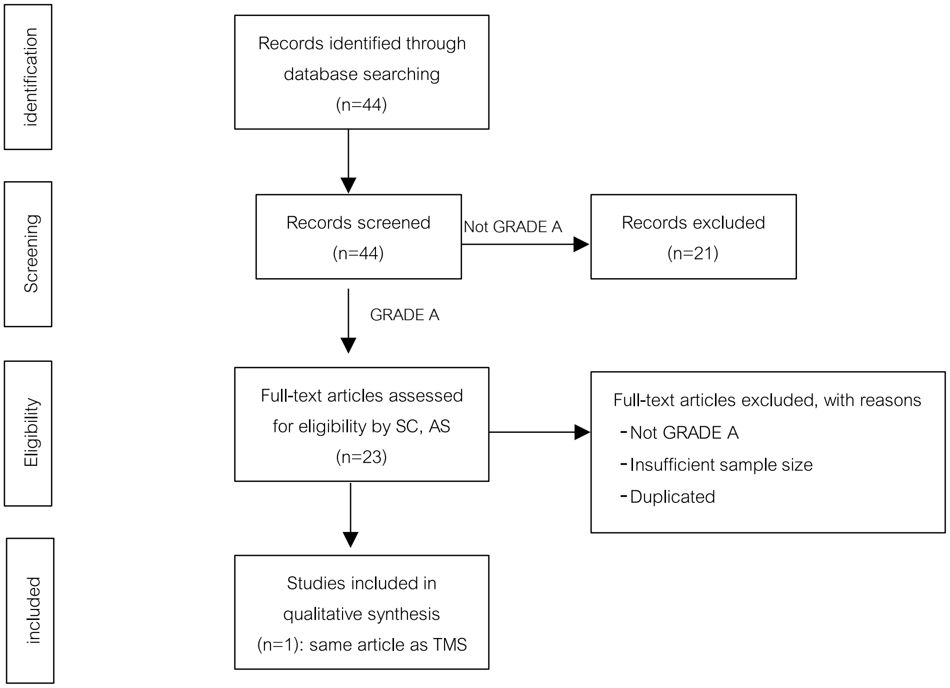

Appendix fig. 7.1: Botulinum toxin injection AND upper extremity AND spasticity AND stroke

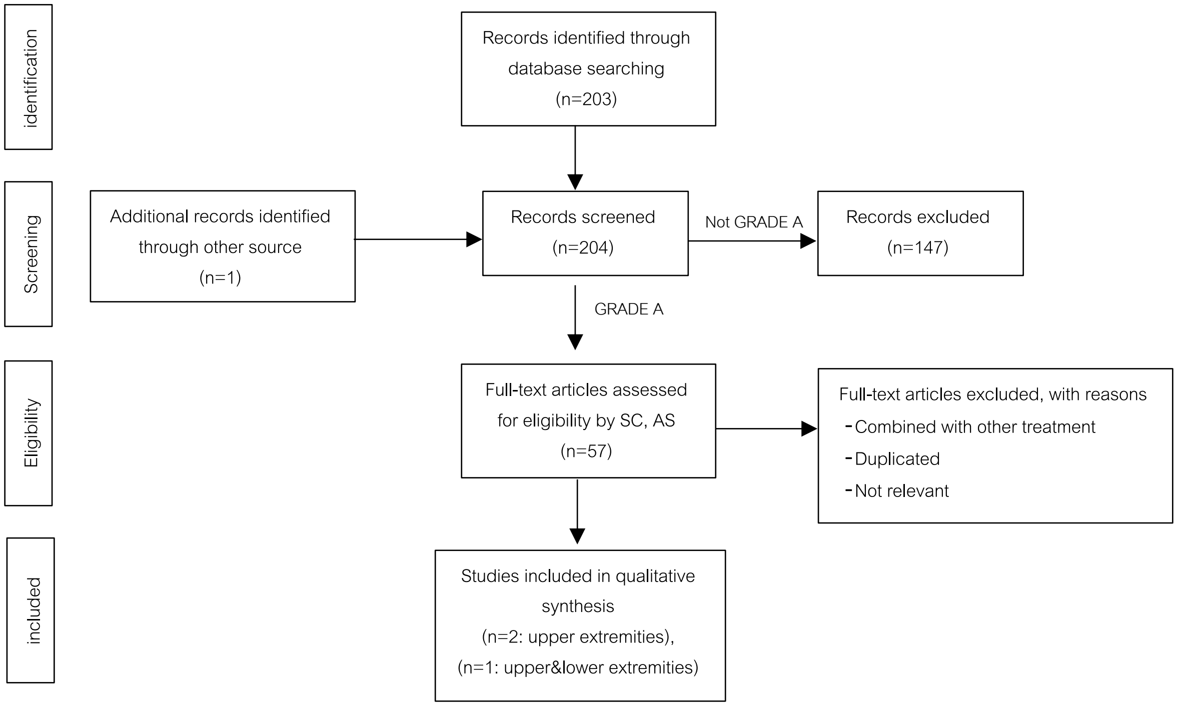

Appendix fig. 7.2: Botulinum toxin injection AND lower extremity AND spasticity AND stroke

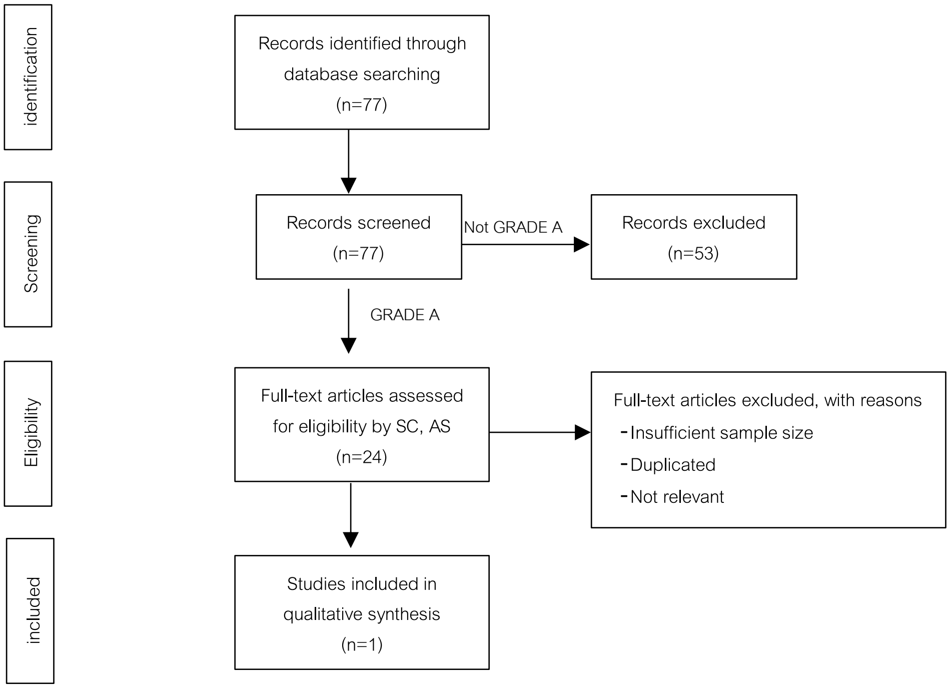

Appendix fig. 8: Dry needling AND spasticity AND stroke

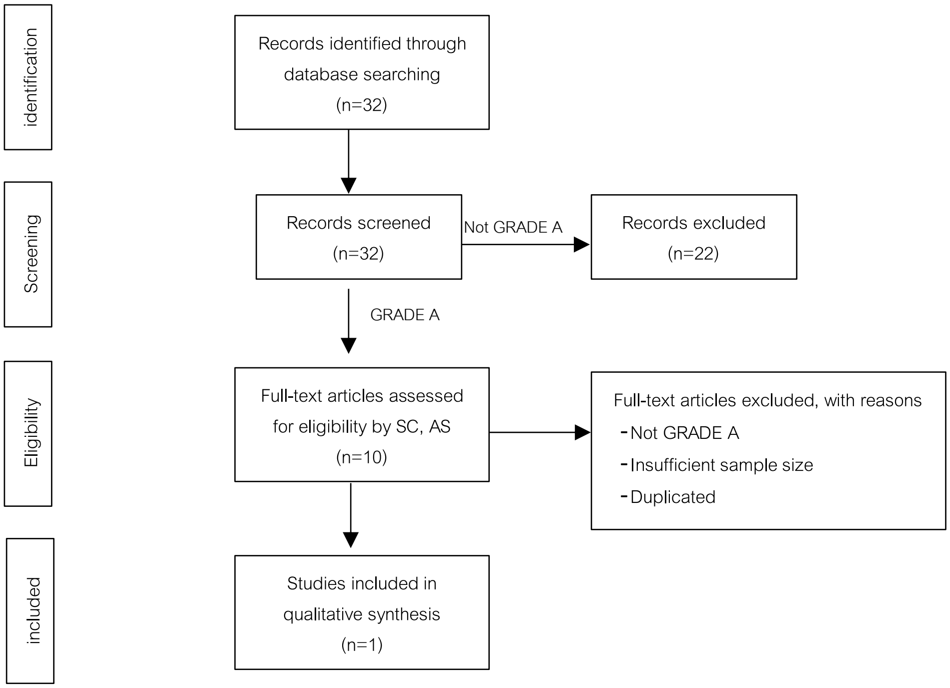

Appendix fig. 9: Intrathecal baclofen AND spasticity AND stroke

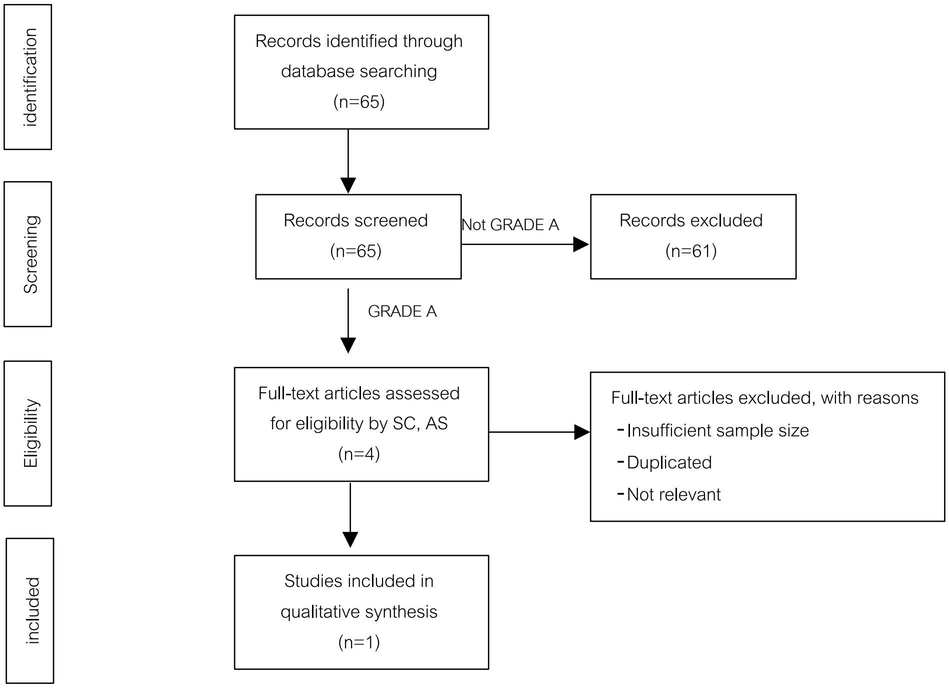

Appendix fig. 10, 11: vibration AND spasticity AND stroke

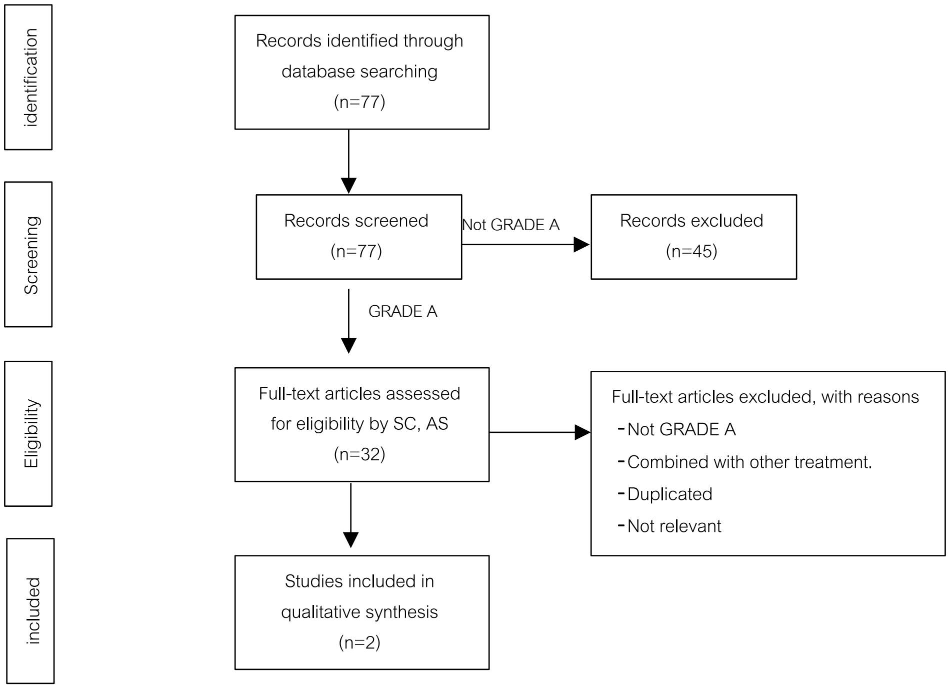

Supplement: Supplementary file 1 [file toxins-16-00098-s001.zip › supplementary-File S1-PRISMA.pdf]
